# Chromosomal variation among populations of a fungus-farming ant: implications for karyotype evolution and potential restriction to gene flow

**DOI:** 10.1186/s12862-018-1247-5

**Published:** 2018-09-21

**Authors:** Danon Clemes Cardoso, Jürgen Heinze, Mariana Neves Moura, Maykon Passos Cristiano

**Affiliations:** 1 Departamento de Biodiversidade, Evolução e Meio Ambiente/ICEB, Universidade Federal de Ouro Preto, Campus Morro do Cruzeiro, Ouro Preto, Minas Gerais 35400-000 Brazil; 2 Zoology/Evolutionary Biology, Universität Regensburg, Universitätstrasse 31, D-93040 Regensburg, Germany; 3 Programa de Pós-graduação em Ecologia, Universidade Federal de Viçosa, Viçosa, Minas Gerais 36570-000 Brazil

**Keywords:** Centromere, Karyotype length, Gene flow, *Trachymyrmex holmgreni*, Formicidae

## Abstract

**Background:**

Intraspecific variation in chromosome structure may cause genetic incompatibilities and thus provides the first step in the formation of species. In ants, chromosome number varies tremendously from 2n = 2 to 2n = 120, and several studies have revealed considerable variation in karyotype within species. However, most previous studies were limited to the description of chromosome number and morphology, and more detailed karyomorphometric analyses may reveal additional, substantial variation. Here, we studied karyotype length, genome size, and phylogeography of five populations of the fungus-farming ant *Trachymyrmex holmgreni* in order to detect potential barriers to gene flow.

**Results:**

Chromosome number and morphology did not vary among the five populations, but karyotype length and genome size were significantly higher in the southernmost populations than in the northern populations of this ant. Individuals or colonies with different karyotype lengths were not observed. Karyotype length variation appears to result from variation in centromere length.

**Conclusion:**

*T. holmgreni* shows considerable variation in karyotype length and might provide a second example of centromere drive in ants, similar to what has previously been observed in *Solenopsis* fire ants. Whether this variation leads to genetic incompatibilities between the different populations remains to be studied.

**Electronic supplementary material:**

The online version of this article (10.1186/s12862-018-1247-5) contains supplementary material, which is available to authorized users.

## Background

Differences in chromosome number, form, and structure may result in genetic incompatibilities, which restrict gene flow among different lineages within a species [1–4], strengthen reproductive isolation among incipient species [5, 6], and prevent hybridization [7, 8]. Spontaneously arising chromosomal rearrangements may accumulate and spread to fixation via genetic drift or selection in allopatric populations. Interpopulation mating between individuals with different underdominant mutations may lead to sterile hybrid offspring [1–8]. Alternatively, chromosome rearrangements can reduce gene flow by suppressing recombination [5, 9]. For example, inversion polymorphisms are associated with the sympatric formation of host races in the apple maggot fly, *Rhagoletis pomonella* [10], and chromosomal rearrangements underlie the divergence of wing-pattern morphs in *Heliconius* butterflies [11].

Ants (Formicidae) with their huge variation in chromosome number from 2n = 2 to 2n = 120 [12] might provide good models to investigate the role of chromosomal variation in speciation. Previous studies have shown that interspecific chromosomal variation differs among ant lineages [12–14]: clades that appear to have retained ancestral traits, such as the poneromorph subfamilies, often show large differences in chromosome number and even variation within populations [12, 15]. In contrast, chromosome numbers appear to be more stable in more derived ant lineages, such as leafcutter ants [16]. Karyotypes differ between species due to Robertsonian rearrangements, inversions, and translocations ([12, 17], and in a number of genera chromosome mutations have been suggested to be involved in speciation (e.g., [12, 18]).

Previous studies have often been limited to the description of chromosome number and morphology, and there is a lack of comprehensive cytogenetic studies. Structural chromosome variation, which does not change chromosome number, is in general more difficult to detect but might nevertheless lead to genetic mismatches [12, 19]. Detailed karyomorphometric studies would therefore be highly informative to better understand chromosomal variation and possible barriers of gene flow in ants [12, 20, 21]. Of particular relevance is variation in the length of the centromeres, highly repetitive DNA sequences that link pairs of sister chromatids. Differences in centromere length may result from centromeric chromatin enhancing the frequency of mutations frequencies and inhibiting DNA repair [22] or from “centromere drive,” i.e., competition among selfish genetic elements for transmission to the oocyte during female meiosis [23, 24]. In any case, the rapid evolution of the DNA and protein components of centromeric chromatin may be responsible for the reproductive isolation of emerging species [9, 23, 24]. Based on the observation of extremely long centromeres in several species of *Solenopsis* fire ants, it was suggested that centromere drive is more common in Hymenoptera [25] and could provide an additional barrier to gene flow between populations.

Here we use a karyomorphometrical analysis to characterize the karyotype of the fungus-growing ant *Trachymyrmex holmgreni* Wheeler, 1925 from five geographically distinct populations. These chromosome analyses were complemented by an estimation of genome size differences by flow cytometry and a phylogeographic analysis of the studied populations. We document inter-population variation of karyotype length that match the model of centromere drive and may be promoting the isolation of populations.

## Results

### Karyotype analysis and chromosome banding

**Fig. 1:**
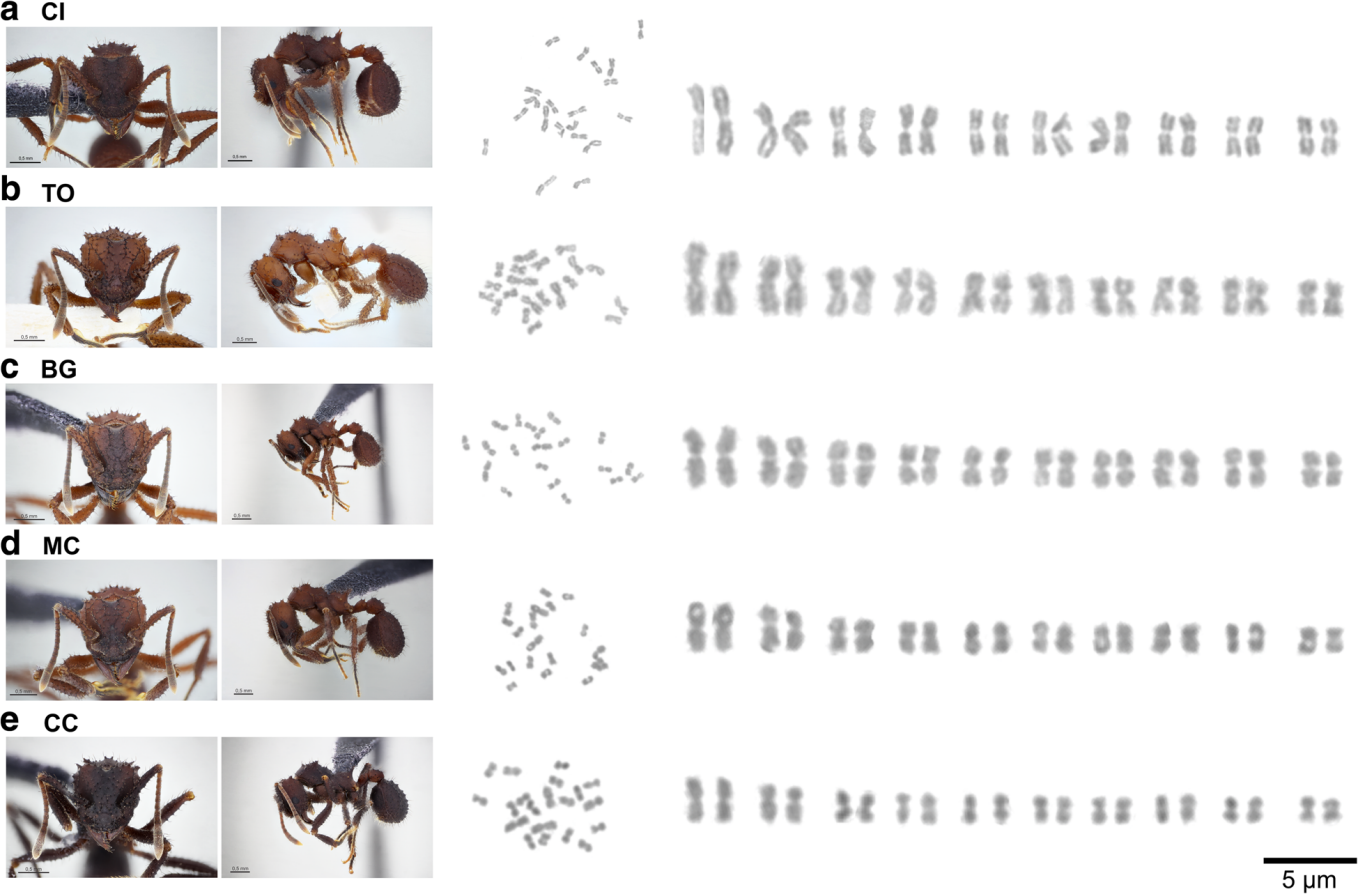
Conventional staining of mitotic cells of the ant *Trachymyrmex holmgreni.* Images of workers, metaphases, and diploid karyotypes of populations of *T. holmgreni*: (**a**) CI – Cidreira, (**b**) TO – Torres, (**c**) BG – Balneário Gaivota, (**d**) MC – Morro dos Conventos, and (**e**) CC – Cachoeira do Campo

**Fig. 2:**
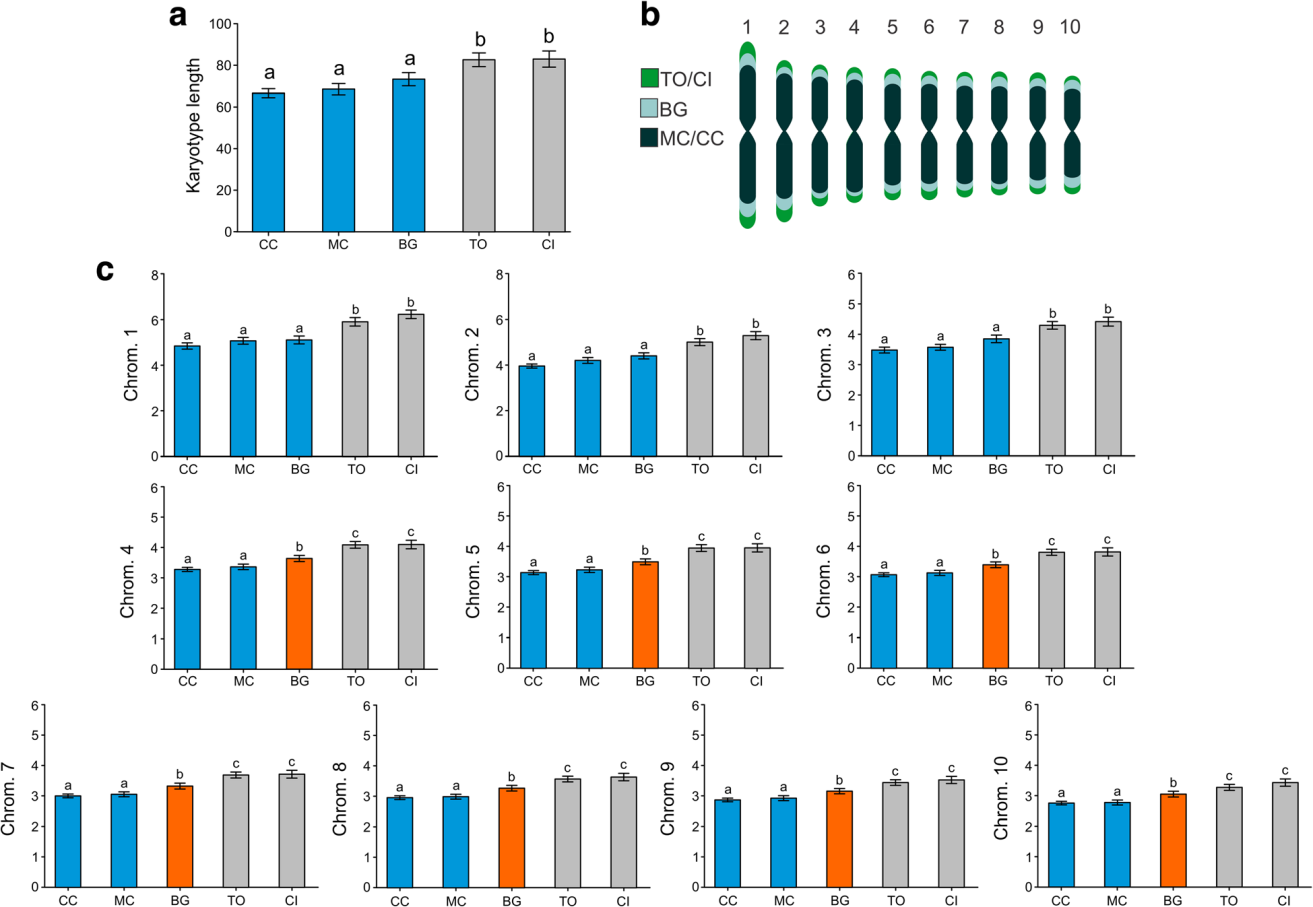
Statistical karyomorphometry of *Trachymyrmex holmgreni* populations. **a** Average karyotype length variation among populations. The sum of mean chromosome lengths (in μm) varied significantly among populations (GLM: Deviance _(3,36)_ = 4284.7; *p* = 0.0004). **b** Idiograms showing the relative contributions of each chromosome to the variation of karyotype length among populations. **c** Length of individual chromosomes (in μm) in the five populations. Different letters and colors indicate statistically different contrasts and significant different contributions of each homologue to the variation of total karyotype length between populations

The karyotype of *T. holmgreni* was 2n = 20 (*n* = 10), with all chromosomes being metacentric, which represents the karyotype formula  2K = 20 M and a diploid number of the arms 2AN = 40 (Fig. 1, Additional file 1: Tables S1-S5). There was no numerical or morphological variation among the populations studied, not even between the geographically most distant populations of Cidreira (CI) and Cachoeira do Campo (CC). Surprisingly, karyotype length (the sum of each averaged chromosome length in a particular set) varied significantly among populations (GLM: Deviance_(4,45)_ = 4284.7; *p* = 0.0004) (all pairwise differences *p* < 0.05), except for the populations of Morro dos Conventos (MC), Balneário Gaivota (BG), and CC, which did not differ (*p* > 0.05; Fig. 2a). In the populations of CI, Torres (TO) and BG, the chromosome sizes ranged from 6.29 ± 0.82 μm to 3.18 ± 0.45 μm, 6.06 ± 0.87 μm to 3.40 ± 0.54 μm, and 5.30 ± 0.78 μm to 3.00 ± 0.46 μm with mean karyotype lengths of 83.06 μm, 82.72 μm and 73.38 μm, respectively (Table 1, Additional file 1: Tables S1-S5). However, in the populations of MC and CC, the sizes of the chromosomes ranged from 5.25 ± 0.69 μm to 2.70 ± 0.39 μm and from 4.87 ± 0.60 μm to 2.62 ± 0.25 μm, with a total length of 68.63 μm and 66.08 μm, respectively (Table 1, Additional file 1: Tables S1-S5). Comparing each homologous chromosome across populations revealed that each chromosome individually contributed for variation in karyotype length in the CI and TO populations and seven pairs contributed to the variation in the BG population (Fig. 2b, c): Chromosome 1 (GLM: Deviance_(4,95)_ = 53.253, *p* < 0.001); Chromosome 2 (GLM: Deviance_(4,95)_ = 36.995, *p* < 0.001); Chromosome 3 (GLM: Deviance_(4,95)_ = 27.157, *p* < 0.001); Chromosome 4 (GLM: Deviance_(4,95)_ = 20.856, *p* < 0.001); Chromosome 5 (GLM: Deviance_(4,95)_ = 19.820, *p* < 0.001); Chromosome 6 (GLM: Deviance_(4,95)_ = 18.241, *p* < 0.001), Chromosome 7 (GLM: Deviance_(4,95)_ = 17.439, *p* < 0.001); Chromosome 8 (GLM: Deviance_(4,95)_ = 16.236, *p* < 0.001); Chromosome 9 (GLM: Deviance_(4,95)_ = 15.243, *p* < 0.001) and Chromosome 10 (GLM: Deviance_(4,95)_ = 16.302, *p* < 0.001). All measurements had low variability and all individual CV values were within one standard deviation of the mean CV. The CVs were not significantly different (GLM: df = 1, deviance = 0.0339, *p* = 0.67), thus the averaged measurements of chromosomes represent a good and stable value of *T. holmgreni* karyotypes.

**Table 1:** Karyomorphometric analyses of the chromosomes of *T. holmgreni* analyzed in the present study. Chromosomes total length (TL) and Karyotype length (KL) for each sampled site

Chromosome	CI TL(μM)	TO TL(μM)	BG TL(μM)	MC TL(μM)	CC TL(μM)
1	6.29 ± 0.82	6.06 ± 0.87	5.30 ± 0.78	5.25 ± 0.69	4.87 ± 0.60
1	6.06 ± 0.83	5.77 ± 0.81	4.93 ± 0.77	4.90 ± 0.61	4.58 ± 0.63
2	5.40 ± 0.81	5.10 ± 0.62	4.48 ± 0.71	4.31 ± 0.58	4.17 ± 0.41
2	5.03 ± 0.76	4.92 ± 0.59	4.34 ± 0.70	4.10 ± 0.57	3.80 ± 0.37
3	4.48 ± 0.58	4.35 ± 0.69	3.90 ± 0.61	3.61 ± 0.43	3.42 ± 0.48
3	4.28 ± 0.51	4.23 ± 0.66	3.80 ± 0.54	3.53 ± 0.45	3.34 ± 0.40
4	4.13 ± 0.51	4.11 ± 0.66	3.67 ± 0.46	3.42 ± 0.40	3.28 ± 0.31
4	4.04 ± 0.50	4.05 ± 0.63	3.59 ± 0.46	3.30 ± 0.42	3.21 ± 0.32
5	3.98 ± 0.51	3.98 ± 0.62	3.53 ± 0.44	3.26 ± 0.41	3.20 ± 0.32
5	3.91 ± 0.51	3.92 ± 0.61	3.46 ± 0.44	3.21 ± 0.40	3.14 ± 0.29
6	3.84 ± 0.46	3.85 ± 0.62	3.41 ± 0.44	3.16 ± 0.40	3.07 ± 0.28
6	3.76 ± 0.45	3.77 ± 0.61	3.38 ± 0.43	3.10 ± 0.36	3.04 ± 0.29
7	3.71 ± 0.45	3.73 ± 0.60	3.34 ± 0.45	3.07 ± 0.36	3.00 ± 0.28
7	3.64 ± 0.45	3.70 ± 0.58	3.31 ± 0.44	3.04 ± 0.38	2.99 ± 0.29
8	3.59 ± 0.45	3.67 ± 0.57	3.28 ± 0.43	3.00 ± 0.37	2.96 ± 0.28
8	3.52 ± 0.40	3.60 ± 0.56	3.26 ± 0.43	2.98 ± 0.36	2.92 ± 0.29
9	3.46 ± 0.40	3.56 ± 0.55	3.18 ± 0.44	2.94 ± 0.38	2.89 ± 0.29
9	3.41 ± 0.39	3.50 ± 0.53	3.13 ± 0.41	2.91 ± 0.37	2.80 ± 0.26
10	3.35 ± 0.40	3.45 ± 0.55	3.09 ± 0.44	2.85 ± 0.34	2.77 ± 0.27
10	3.18 ± 0.45	3.40 ± 0.54	3.00 ± 0.46	2.70 ± 0.39	2.62 ± 0.25
∑(KL)	83.06 μM	82.72 μM	73.38 μM	68.63 μM	66.08 μM

**Fig. 3:**
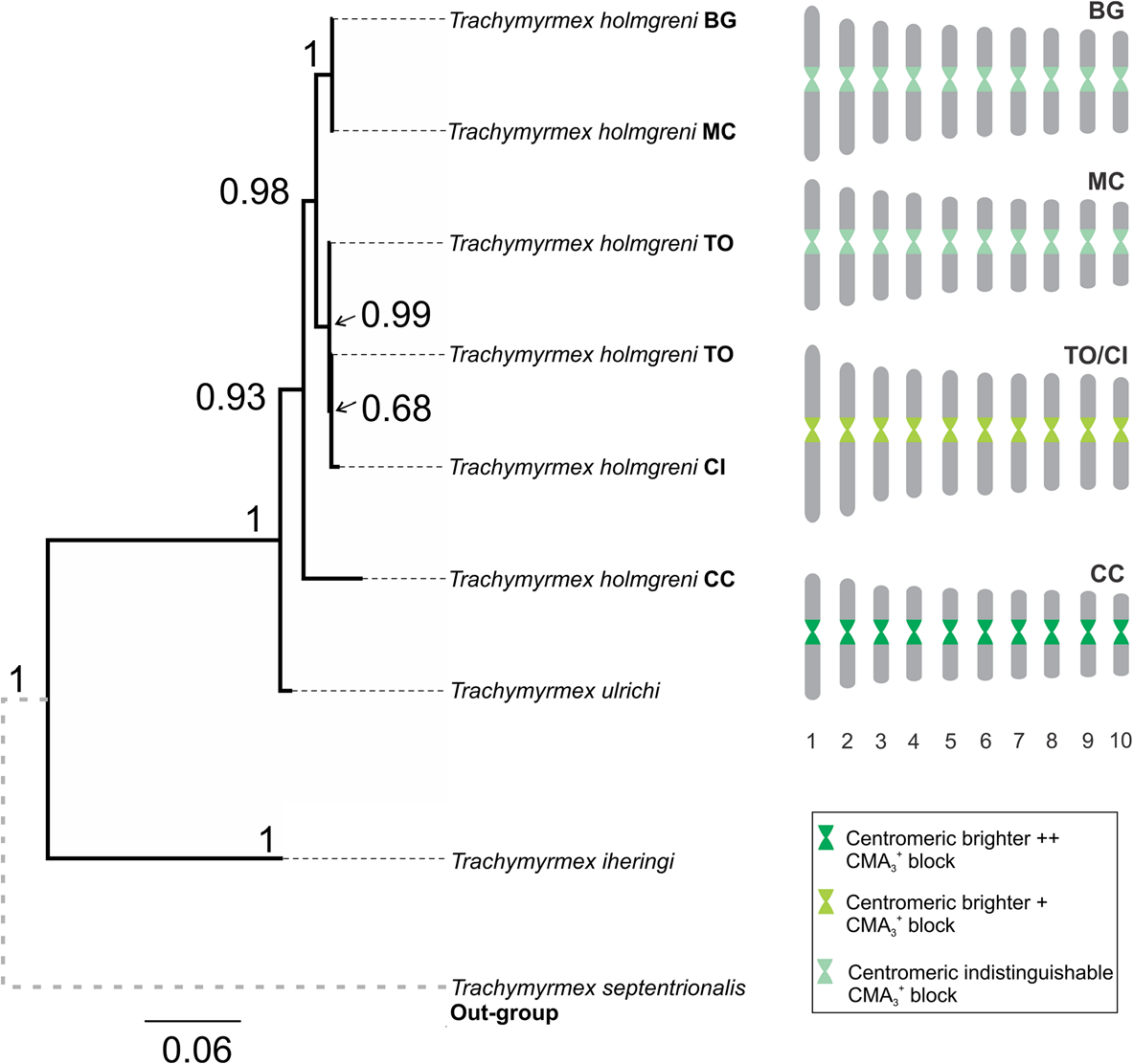
Phylogenetic relationships among *T. holmgreni* populations and ideograms depicting the CMA_3_ patterns of chromosomes. Phylogenetic tree obtained by a Bayesian analysis of mtDNA sequences of COI-tRNAleu-COII of *T. holmgreni* and outgroups (*Trachymyrmex* spp.). The numbers at the nodes indicate the posterior probabilities (PP) from the Bayesian analysis. For each population, an ideogram based on karyomorphometric data, indicates the different brightness observed after fluorochrome staining

**Fig. 4:**
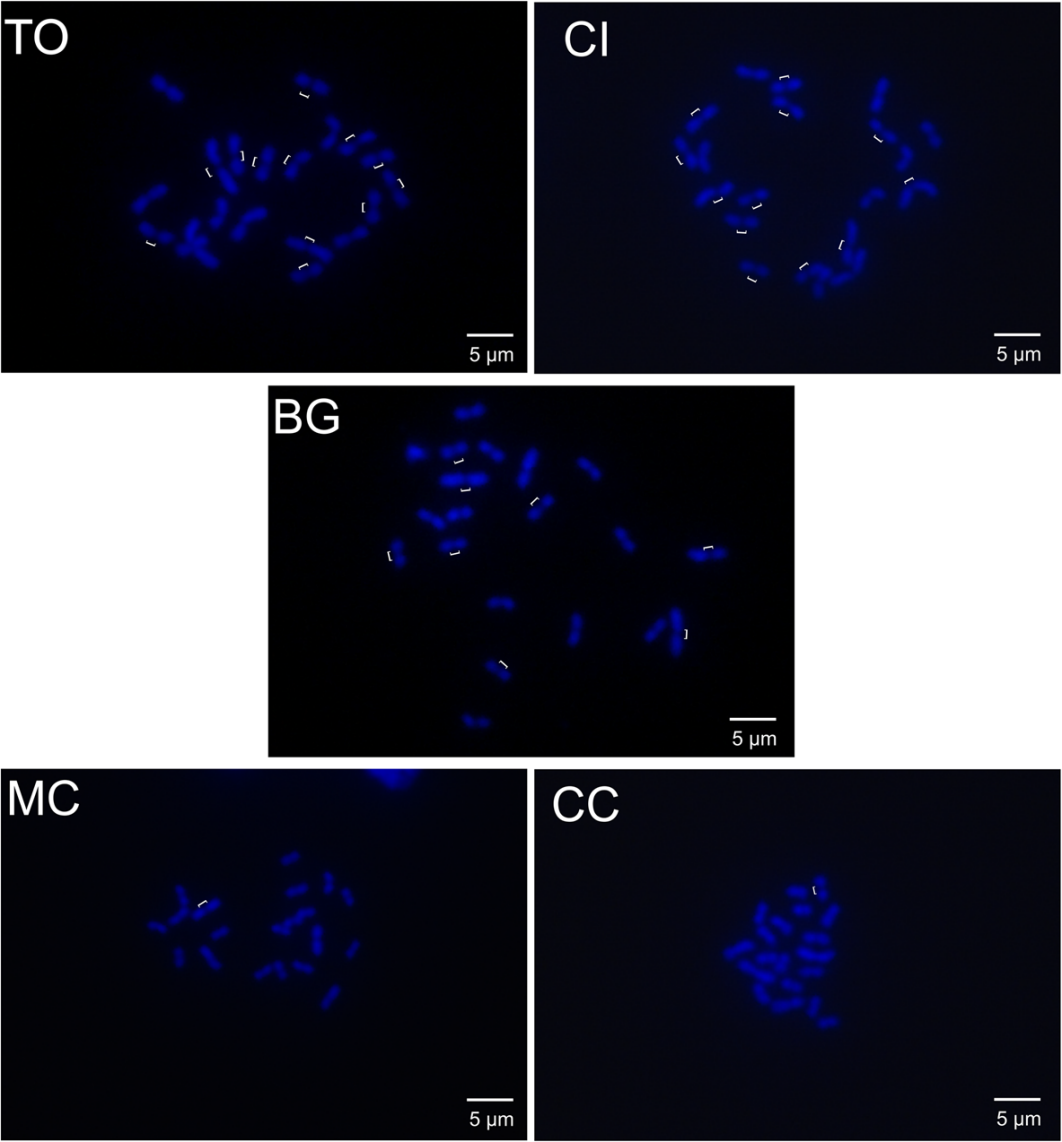
Centromere variation in *Trachymyrmex holmgreni populations*. The centromere was revealed by staining the metaphases with DAPI only. The white bars indicate the distinctly longer centromeres in the populations CI, TO, and BG and for comparison the shorter centromeres in one chromosome each in MC and CC

Heterochromatin was evident as positive blocks restricted to the centromeric regions and its location did not differ among populations (Additional file 2: Figure S1). Sequential fluorochrome staining revealed in all chromosome pairs positive GC-rich blocks (CMA_3_^+^) that coincided with the C–bands, indicating that the heterochromatin is GC-rich. DAPI showed a general uniform banding pattern non-concurrent with the CMA_3_^+^ blocks (Additional file 2: Figure S1)*.* In addition*,* we could observe variation in the intensity of the CMA_3_^+^ blocks between populations (see Fig. 3, Additional file 3: Figure S2). In the CC population, chromosomes had prominent CMA_3_^+^ blocks on the centromeres that were evident even in the interphase nucleus. This pattern was never observed in the remaining populations and represents centromeres in interphase nuclei (Additional file 3: Figure S2). CMA_3_^+^ blocks were slightly brighter in TO, similar to CC. Statistical analysis revealed that each homologue contributes to the variation in mean karyotype length among populations, reaching to differences in total chromosome length of ≥10 μm (Table 1). DAPI-staining revealed that the centromeric interval varied among chromosomes and between karyotypes with smaller and larger karyotype length (Fig. 4), suggesting that the differences in karyotype length are due to variation in centromere length.

### Genome size estimation by flow cytometry

**Fig. 5:**
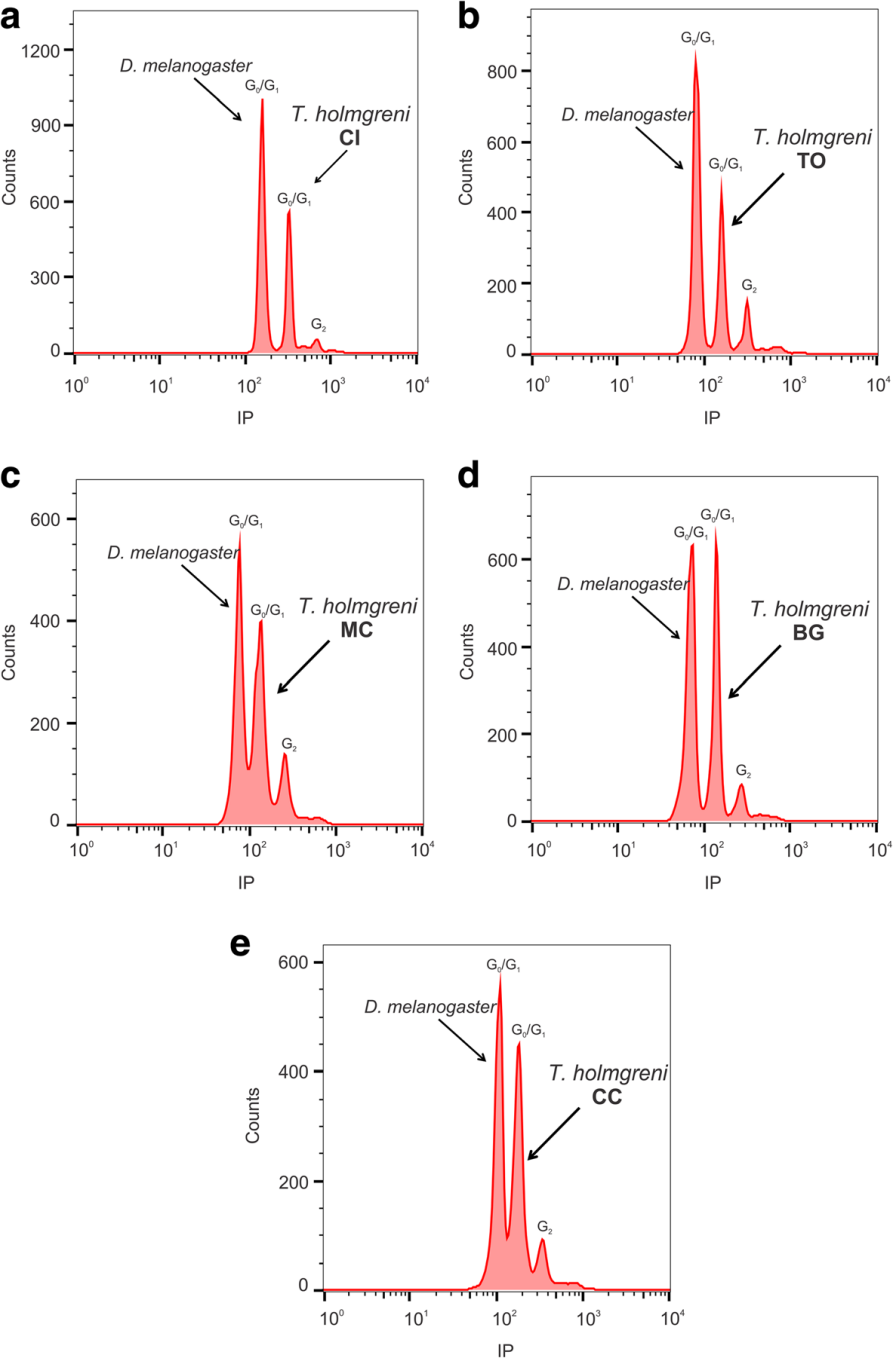
Genome size of *Trachymyrmex holmgreni* populations estimated by flow cytometry. **a** CI – Cidreira 2C = 0.70 ± 0.004 pg, (**b**) TO – Torres 2C = 0.70 ± 0.001 pg, (**c**) BG – Balneário Gaivota 2C = 0.70 ± 0.003 pg, (**d**) MC – Morro dos Conventos 2C = 0.62 ± 0.004 pg, and (**e**) CC – Cachoeira do Campo 2C = 0.62 ± 0.002 pg.

The 1C-value of *T. holmgreni* ranged from 0.30 to 0.35 pg (293.4 to 342.3 Mbp). Mean genome size varied significantly between populations (GLM: Deviance_(3,62)_ = 0.020538, *p* < 0.001) and the contrast analysis distinguished CC (mean ± SD: 0.31 ± 0.002 pg or 303.18 Mbp) and MC (0.31 ± 0.004 pg, 303.18 Mbp) from BG (0.35 ± 0.003 pg, 342.3 Mbp), TO (0.35 ± 0.001 pg, 342.3 Mbp), and CI (0.35 ± 0.004 pg, 342.3 Mpb, Fig. 5). These results suggest that the genomes are 0.04 pg or 39.12 Mbp larger in the two populations with longer chromosomes (BG, TO and CI) than in the populations with shorter chromosomes (CC and MC).

### Phylogenetic analysis

To describe the relationship among colonies from the five populations we performed a phylogenetic analysis of COI-tRNAleu-COII haplotypes using Bayesian inference. Our tree shows that the colonies from BG plus MC form a monophyletic clade (posterior probability PP = 1) and are more closely related to the clade TO plus CI (PP = 0.99) than to the distant CC population (PP = 0.93). This matches the results of karyomorphometry: the most genetically and geographically most distant population showed the most intense CMA_3_^+^ blocks on the centromeres (Fig. 3).

## Discussion

Our study revealed that ants from geographically and genetically distant populations of the ant *Trachymyrmex holmgreni* have similar chromosome number and morphology (2n = 20 and  2K = 20 M), suggesting chromosomal stability. Nevertheless, a karyomorphometrical approach as described by Cristiano et al. [21] and the estimation of genome size indicated considerable inter-population variation in karyotype length. Similar length polymorphisms are known from other ant species [20] (see also Cardoso and Cristiano *in preparation*), but they typically do not involve stable inter-population variation of all chromosomes. Karyotype length appears to be invariable within populations of *T. holmgreni,* and each chromosome contributes to the length variation of total karyotype length (see Fig. 2).

Overall, polymorphisms in chromosome size can be consequences of changes in heterochromatic regions composed mainly of repetitive DNA, e.g. [26]. In *T. holmgreni* we did not find evidence for large variation in the distribution of heterochromatin, which was clearly visible and restricted to the centromeric region. The difference in karyotype length appears to be related to the evolution of longer centromeres, as evidenced by the long negative blocks of DAPI staining along the centromeric region. Additional evidence for centromere differences comes from the intensity variation of the CMA_3_^+^ blocks, which directly reflects differences in the richness of CG nucleotides [27] and may point to marked changes in the nucleotide composition of the centromeric satellite DNA of *T. holmgreni*.

Centromere drive leads to a rapid evolution of centromeric satellite DNA and may be responsible for the reproductive isolation of emerging species [9, 23, 24]. In *Solenopsis* fire ants, centromere drive has been suggested to increase the number of copies of *CenSol*, the major centromere satellite DNA repeat, and thus to lead to the evolution of extremely long centromeres in certain species [25]. The variation in centromere length in *T. holmgreni* might provide a second example of centromere drive. According to the phylogeny of our samples, the southern populations TO and CI with the longest karyotype length are nested within the populations with shorter karyotype lengths (see Fig. 3), which matches the model of runaway centromere expansion [25].

Marked differences in centromere length might generally act as a barrier to gene flow and could promote reproductive isolation [9, 23, 24]. Unfortunately, the notorious unwillingness of most ant sexuals to mate in the lab will make it difficult to investigate whether karyotype length variation is already associated with genetic incompatibility. However, hybrid colonies showing homologous chromosomes with different sizes were not found among the analyzed 56 colonies of *T. holmgreni*. While in the related ant species *Trachymyrmex fuscus* [20] and *Mycetophylax simplex* (Cardoso and Cristiano *in preparation*) homologues with different sizes are able to form bivalents in meiosis (see also [28]), the absence of hybrids in *T. holmgreni* may reflect both the geographic isolation of the populations and a potential incompatibility of the different chromosome sizes. Nevertheless, in the absence of firm data about genetic incompatibilities between *T. holmgreni* with different karyotype length our study remains limited to the description of intraspecific variation in chromosome length.

## Conclusion

The results obtained in the present study on karyotype traits across *T. holmgreni* populations showed changes in their fine structure, which might be the first steps of chromosome evolution. The application of a standardized karyomorphometrical approach coupled with a statistical analysis is important to unveil hidden chromosomal variation. The differences in karyotype and chromosome lengths are consistent with the recent proposed model of centromere expansion in ants and might be a common mechanism of karyotype change in Formicidae.

## Methods

### Sampled colonies

Colonies of *T. holmgreni* were sampled in April and November of 2016 and in March of 2018 in five different localities: Cidreira, state of Rio Grande do Sul (CI, S30°08′39″ W50°12′19″, 4 colonies), Torres, state of Rio Grande do Sul (TO; S29°24′01″ W49°46′33.4″, 14 colonies), Balneário Gaivota, state of Santa Catarina (BG; S29°11′42.23″ W49°36′30.9″, 20 colonies), Morro dos Conventos, Santa Catarina (MC; S28°56′07.9″ W49°21′28.29″, 15 colonies), and Cachoeira do Campo, state of Minas Gerais (CC; S20°20′56.5″ W43°40′20.7″, 3 colonies). The southern localities are coastal plain dunes, with MC and TO about 35 km to the North and South of BG, and CI about 105 km south of TO (Additional file 4: Figure S3). The population of CC is situated inland approximately 1500 km north of the southern sampling sites. It is situated in a transition zone between Atlantic and “Cerrado” (Brazilian savannas) and consists of open and shrubby areas similar to the coastal sand dune areas in the other populations. While we cannot completely exclude gene flow between the neighboring sites MC, BG, and TO, the patchy occurrence of suitable habitat and presumed low dispersion capacity of *T. holmgreni* makes it unlikely that the samples from these sites all belong to the same population.

Nests were identified by the presence of a tower of straw and a circular mound of sand (see also [29]). Then, the colonies were excavated and transferred to the Laboratório de Genética Evolutiva e de Populações of the Universidade Federal de Ouro Preto, where they were maintained following the protocol described by Cardoso et al. [30] to obtain brood to be used in the present study. All colonies sampled in 2016 were kept alive until 2017, the colonies from Cidreira sampled 2018 were still maintained in the lab at the time of manuscript preparation.

### Karyotype characterization and chromosome structure

We analyzed at least 10 larvae from each of the 56 sampled colonies, totaling 560 samples. Metaphase chromosomes were obtained from cerebral ganglia of prepupae using a protocol by Imai et al. [31], modified following Cardoso et al. [32]. The metaphases were evaluated qualitatively under a phase-contrast microscope and the ≥30 best slides per sampling site with well-spread chromosomes were used to determine the number and morphology of chromosomes after conventional staining with Giemsa. C-band staining was used to determine the distribution pattern of heterochromatin, as described by Sumner [33], with modifications proposed by Pompolo & Takahashi [34]. Sequential staining with fluorochromes was performed using chromomycin A3/distamycin A/4′-6-diamidino-2-phenylindole (CMA_3_/DA/DAPI) to characterize regions rich in CG and AT base pairs, respectively [35]. The metaphases were photographed under a light microscope and the Zeiss AxioImager Z2 epifluorescence microscope with integrated digital camera (AxioCam Mrc). The fluorochrome slides were analyzed using GFP filters (450 to 480 nm) for CMA_3_ and DAPI (330 to 385 nm) for DAPI. Sequential fluorochrome staining and C-banding could not be done with samples from CI because of the lack of a sufficient number of larvae. Chromosome morphology was classified following the nomenclature proposed by Levan et al. [36], which uses the centromere position and the relative arm lengths to classify them as acrocentric (A), subtelocentric (ST), submetacentric (SM) and metacentric (M).

Karyomorphometrical analyses were carried out on the 10 best-spread metaphases with chromosome integrity from each population according to the procedures described by Cristiano et al. [21]. Briefly, we measured on Image Pro Plus ® software (Media Cybernetics, Rockville, MD) each individual chromosome from centromere to the end of the long arm (L) and the short arm (S), and also the total chromosome length (TL). Chromosome length was averaged across the 10 individuals measured from each colony. The summed length of all chromosomes is given as karyotype length (KL). Differences in the length of centromeres were determined by staining metaphases with DAPI following Huang et al. [25].

We evaluated arm ratio (*r* = L/S), chromosome length (RL) of each chromosome relative to the sum of all chromosome lengths in the particular sample (TL × 100/∑TL), and asymmetry index (∑long arms/∑total length × 100). The coefficient of variation (CV) was used to quantify the degree of variation among measurements for each specimen and then validate our measurements (Additional file 5: Table S6).

We analyzed differences in the CV, TL, and mean KL across specimens and populations by generalized linear models (GLM) as implemented in R v. 3.2.0 by R Development Core Team. For all GLM models, when significant differences were observed among populations, we carried out an analysis of contrast at a significance level of 5% (5%) to determine the different groups using R. Thus, if the level of aggregation was not significant and did not alter the deviance explained by the null model, the levels were pooled and the model was adjusted, allowing us to determine which populations differed from each other.

### Genome size estimation by flow cytometry

Genome size (in picogram, pg) was estimated by flow cytometry in individuals from four colonies from CI, three colonies from TO, four colonies from BG, two colonies from MC, and two colonies from CC following the protocol established by Moura et al. (unpublished data). Briefly, the heads of adult workers and the internal standard (*Drosophila melanogaster*) were cut with a cutting blade and immersed in 100–300 μL of Galbraith buffer and ground to release the cell nuclei. Subsequently, 600 μL of the buffer were added, filtered through a 40 μm nylon mesh and stained by adding 6.5 μL of propidium iodide solution and 3.5 μl RNAse. The samples were stored at 4 °C in the dark and analyzed within 1 h after preparation.

The analyses were performed on a FACSCalibur (BD Biosciences, San José, USA) cytometer at Universidade Federal de Ouro Preto, equipped with a laser source (488 nm) and the histograms were obtained by the BD Cell Quest software. For each sample, at least 10,000 nuclei were analyzed regarding their relative fluorescence intensity. Three independent replicates (three individuals per colony) were conducted and histograms with a coefficient of variation above 5% were rejected. Histograms were analyzed using the Flowing 2.5.1 software (http://www.flowingsoftware.com). The genome size of each specimen was calculated using the 1C-value (0.18 pg) of *Drosophila melanogaster* and the values were obtained according the equation given by Doležel and Bartos [37] and subsequently converted to megabasepairs (1 pg = 978 Mbp).

### DNA extraction, PCR amplification, sequencing

We extracted genomic DNA from one worker from two colonies per population, following a modified phenol-chloroform protocol [38]. Mitochondrial sequences were obtained for the COI-tRNA Leucine-COII region using the primers C1-J- 2195 (alias CO1-RLR) (5′-TGATTTTTTGGTCATCCAGAAGT-3′) and C2-N-3661 (alias Barbara) (5′- CCACAAATTTCTGAACATTGACCA-3′), following Seal et al. [39]. Polymerase chain reaction (PCR) was performed using 2 U of GoTaq® Flexi DNA Polymerase (Promega), dNTPs (0.25 mM each), MgCl_2_ (2.5 mM), reaction buffer (1×), a pair of primers (0.48 μM each) and 1 μL of DNA, in a final volume of 25 μL. The amplification reaction included 2 min denaturation at 94 °C, followed by 35 cycles of 94 °C for 1 min, 55 °C for 1 min, and 72 °C for 1 min, with a final extension at 72 °C for 5 min.

The amplicons were sent to Macrogen Inc., South Korea (www.macrogen.com) and Myleus Inc., Brazil (http://www.myleus.com), purified, and sequenced directly in both directions (forward and reverse) using the same primers as in the amplification reactions. Forward and reverse strands were visually inspected and assembled using the program Geneious v.R8 (Biomatters Ltd., Auckland, New Zealand). Sequences were first translated into amino acid sequences to guarantee the homology of the sites and to exclude the possible presence of stop codons or indels [40]. Thereafter, the nucleotides were aligned using the Muscle implemented in MEGA 7 software [41]. Because of low Phred quality scores, only one sequence was used per population, except for TO.

### Phylogenetic analysis

The alignment comprised sequences of *Trachymyrmex holmgreni* from the five populations, one sample of *Trachymyrmex iheringi* from Araranguá, Santa Catarina state, and one sample of *Trachymyrmex ulrichi* from Laguna, Santa Catarina state (all sequences were deposited in Genbank: MH747644-MH747652). One sequence of *Trachymyrmex septentrionalis* from GenBank was included as outgroup.

Bayesian analysis was conducted for phylogenetic inference using MrBayes 3.2 [42]. PartitionFinder2 [43, 44] was used to estimate the nucleotide substitution model that best fit each gene codon position under Akaike’s information criterion. The Bayesian analyses consisted of two independent runs of 10 million generations each, sampled every 1000 generations and four chains. After discarding the first 25% of MCMC generations as burn-in, tree topologies were summarized in a consensus tree representing 75% of the trees sampled during the 10,000 MCMC generations and visualized using FigTree v1.4 (http://tree.bio.ed.ac.uk/software/figtree). Bayesian posterior probabilities (PP) indicate support for the various nodes.

## Additional files

Additional file 1: **Table S1-S5.** Results from the karyomorphometrical analyses of the *Trachymyrmex holmgreni* populations analyzed in the study. (DOCX 41 kb)

Additional file 2: **Figure S1.** C-banding in worker metaphases of *Trachymyrmex holmgreni* populations**.** (a) TO – Torres (RS), (b) MC – Morro dos Conventos (SC), and (c) Cachoeira do Campo (MG). The arrows point to dark grey heterochromatin blocks. Scale bar   =   5 μm. C-banding was not done in the Cidreira population. (TIF 4265 kb)

Additional file 3: **Figure S2.** Fluorochrome-stained metaphases of *Trachymyrmex holmgreni* from the four studied populations: by columns: CMA_3_, DAPI, merged CMA_3_ over DAPI and merged DAPI over CMA_3_. By rows: (a) TO – Torres, (b) BG – Balneário Gaivota, (c) MC – Morro dos Conventos, and (d) CC – Cachoeira do Campo. Positive GC-rich blocks were observed in all chromosome pairs at the centromere, as represented in the ideograms. Scale bar  =  5 μm. Fluorochrome-staining was not done in the Cidreira population. (TIF 6249 kb)

Additional file 4: **Figure S3.** Map of Brazil showing the collection sites: Cachoeira do Campo – CC, Morro dos Conventos – MC, Balneário Gaivota – BG, Torres – TO and Cidreira – CI. (TIF 5072 kb)

Additional file 5: **Table S6.** Karyomorphometrical analyses of the specimens of *Trachymyrmex holmgreni* from the five populations analyzed in the study. ∑TL: total length; KL mean karyotype length (= ∑TL/2n) ± SD: standard deviation; CV coefficient of variation (= ± SD/KL). All measurements are given in “μM”. (DOCX 57 kb)

## Abbreviations

A: Acrocentric chromosome
BG: Balneário Gaivota beach
CC: Cachoeira do Campo
CI: Cidreira beach
CMA_3_: Chromomycin A3
COI: *Cytochrome oxidase* 1
COII: *Cytochrome oxidase* 2
CV: Coefficient of variation
DA: Distamycin A
DAPI: 4′-6-Diamidino-2-phenylindole
GLM: Generalized linear models
KL: Mean karyotype length
L: Long arm
M: Metacentric chromosome
Mbp: Mega base pairs
MC: Morro dos Conventos beach
*pg*: Picograms
RL: Chromosome relative length
S: Short arm
SM: Submetacentric chromosome
ST: Subtelocentric chromosome
TL: Chromosome total length
TO: Torres beach

## References

[1] Patton JL, Sherwood SW (1983). Chromosome evolution and speciation in rodents. Annu Rev Ecol Syst.

[2] Conflitti IM, Shields GF, Murphy RW, Currie DC (2015). The speciation continuum: ecological and chromosomal divergence in the *Simulium arcticum* complex (Diptera: Simuliidae). Biol J Linn Soc.

[3] Giménez MD, White TA, Hauffe HC, Panithanarak T, Searle JB. Understanding the basis of diminished gene flow between hybridizing chromosome races of the house mouse. Evolution (N Y). 2013:1446–62. 10.1111/evo.12054 23617920

[4] Potter S, Bragg JG, Blom MPK, Deakin JE, Kirkpatrick M, Eldridge MDB, et al. Chromosomal speciation in the genomics era: disentangling phylogenetic evolution of rock-wallabies. Front Genet. 2017;8. 10.3389/fgene.2017.00010. 10.3389/fgene.2017.00010 PMC5301020 28265284

[5] Rieseberg LH (2001). Chromosomal rearrangements and speciation. Trends Ecol Evol.

[6] Ayala FJ, Coluzzi M (2005). Chromosome speciation: humans, *Drosophila*, and mosquitoes. Proc Natl Acad Sci U S A.

[7] Kirkpatrick M, Barton N (2006). Chromosome inversions, local adaptation and speciation. Genetics.

[8] Hoffmann AA, Rieseberg LH (2008). Revisiting the impact of inversions in evolution: from population genetic markers to drivers of adaptive shifts and speciation?. Annu Rev Ecol Evol Syst.

[9] Brown JD, O’Neill RJ (2010). Chromosomes, conflict, and epigenetics: chromosomal speciation revisited. Annu Rev Genomics Hum Genet.

[10] Feder JL, Roethele JB, Filchak K, Niedbalski J, Romero-Severson J (2003). Evidence for inversion polymorphism related to sympatric host race formation in the apple maggot fly, *Rhagoletis pomonella*. Genetics.

[11] Joron M, Frezal L, Jones RT, Chamberlain NL, Lee SF, Haag CCR, Whibley A, Becuwe M, Baxter SW, Ferguson L, Wilkinson PA, Salazar C, Davidson C, Clark R, Quail MA, Beasley H, Glithero R, Lloyd C, Sims S, Jones MC, Rogers J, Jiggins CD, ffrench-Constant RH (2011). Chromosomal rearrangements maintain a polymorphic supergene controlling butterfly mimicry. Nature.

[12] Lorite P, Palomeque T (2010). Karyotype evolution in ants (Hymenoptera: Formicidae), with a review of the known ant chromosome numbers. Myrmecological News.

[13] Cristiano MP, Cardoso DC, Fernandes-Salomão TM (2013). Cytogenetic and molecular analyses reveal a divergence between *Acromyrmex striatus* (Roger, 1863) and other congeneric species: taxonomic implications. PLoS One.

[14] Cardoso DC, Pompolo S das G, Cristiano MP, Tavares MG (2014). The role of fusion in ant chromosome evolution: insights from cytogenetic analysis using a molecular phylogenetic approach in the genus *Mycetophylax*. PLoS One.

[15] Mariano C dos SF, Pompolo S das G, Silva JG, Delabie JHC (2012). Contribution of cytogenetics to the debate on the paraphyly of *Pachycondyla* spp. (Hymenoptera, Formicidae, Ponerinae). Psyche.

[16] Pereira TTP, dos RACCC, Cardoso DC, Cristiano MP (2018). Molecular phylogenetic reconstruction and localization of the (TTAGG)n telomeric repeats in the chromosomes of *Acromyrmex striatus* (Roger, 1863) suggests a lower ancestral karyotype for leafcutter ants (Hymenoptera). Comp Cytogenet..

[17] Imai HT, Crozier RH, Taylor RW (1977). Karyotype evolution in Australian ants. Chromosoma.

[18] Mariano CSF, Delabie JHC, Campos LAO, Pompolo SG (2003). Trends in karyotype evolution in the ant genus *Camponotus* (Hymenoptera: Formicidae). Sociobiology.

[19] Gosálvez J, López-Fernández C, Bella LJ, Butlin RK (1988). Hewitt GH. A hybrid zone between *Chorthippus parallelus parallelus* and *Chorthippus parallelus erythropus* (Orthoptera: Acrididae): chromosomal differentiation. Genome.

[20] Barros LAC, Cardoso de Aguiar HJA, Mariano C dos SF, Delabie JHC, Pompolo S das G (2013). Cytogenetic characterization of the ant *Trachymyrmex fuscus* Emery, 1934 (Formicidae: Myrmicinae: Attini) with the description of a chromosomal polymorphism. Ann la Société Entomol Fr.

[21] Cristiano MP, Pereira TP, Simões LP, Sandoval-Gómez VE, Cardoso DC (2017). Reassessing the chromosome number and morphology of the turtle ant *Cephalotes pusillus* (Klug, 1824) using karyomorphometrical analysis and observations of new nesting behavior. Insects.

[22] Burrack LS, Berman J (2012). Flexibility of centromere and kinetochore structures. Trends Genet.

[23] Henikoff S, Ahamad K, Malik HS (2001). The centromere paradox: stable inheritance with rapidly evolving DNA. Science.

[24] Hughes SE, Hawley RS (2009). Heterochromatin: a rapidly evolving species barrier. PLoS Biol.

[25] Huang Y-C, Lee C-C, Kao C-Y, Chang N-C, Lin C-C, Shoemaker D (2016). Evolution of long centromeres in fire ants. BMC Evol Biol.

[26] Molavi F, Darvish J, Haddad F, Matin MM (2016). Variation of the centromeric heterochromatin region (CHR) in the Iranian house mouse *Mus musculus* Linnaeus, 1758 (Rodentia: Muridae). Caryologia.

[27] Kowalska A, Bozsaky E, Ramsauer T, Rieder D, Bindea G, Lörch T (2007). A new platform linking chromosomal and sequence information. Chromosom Res.

[28] White MJD. Animal cytology and evolution. Cambridge: Cambridge University Press; 1973. p. 963.

[29] Albuquerque EZ, Diehl-Fleig E, Diehl E, Mayhé-Nunes AJ (2018). Sex investment ratios and natural history observations in a population of *Trachymyrmex holmgreni* (Formicidae) in southern Brazil. Insect Soc.

[30] Cardoso DC, Cristiano MP, Tavares MG (2011). Methodological remarks on rearing basal Attini ants in the laboratory for biological and evolutionary studies: overview of the genus *Mycetophylax*. Insect Soc.

[31] Imai HT, Taylor RW, Crosland MWJ, Crozier RH (1988). Modes of spontaneous chromosomal mutation and karyotype evolution in ants with reference to the minimum interaction hypothesis. Japanese J Genet.

[32] Cardoso DC, Cristiano MP, Barros LAC, Lopes DM, Pompolo S das G (2012). First cytogenetic characterization of a species of the arboreal ant genus *Azteca* Forel, 1978 (Dolichoderinae, Formicidae). Comp Cytogenet.

[33] Sumner AT (1972). A simple technique for demonstrating centromeric heterochromatin. Exp Cell Res.

[34] Pompolo SG, Takahashi CS (1990). Chromosome numbers and C-banding in two wasp species of the genusPolistes (Hymenoptera Polistine, Polistini). Insect Soc.

[35] Schweizer D (1980). Simultaneous fluorescent staining of R bands and specific heterochromatic regions (DA-DAPI bands) in human chromosomes. Cytogenet Genome Res.

[36] Levan A, Fredga K, Sandberg AA (2009). Nomenclature for centromeric position on chromosomes. Hereditas.

[37] Doležel J, Bartoš J (2005). Plant DNA flow cytometry and estimation of nuclear genome size. Ann Bot.

[38] Sambrook J, Russell D (2001). Molecular cloning: a laboratory manual.

[39] Seal JN, Kellner K, Trindl A, Heinze J (2011). Phylogeography of the parthenogenic ant *Platythyrea punctata*: highly successful colonization of the West Indies by a poor disperser. J Biogeogr.

[40] Cristiano MP, Cardoso DC, Fernandes-Salomão TM. Could pseudogenes be widespread in ants? Evidence of *numts* in the leafcutter ant *Acromyrmex **striatus* (Roger, 1863) (Formicidae: Attini). Comptes Rendus Biologies. 2014;337(2):78–85. 10.1016/j.crvi.2013.11.007 24581801

[41] Kumar S, Stecher G, Tamura K. MEGA7: molecular evolutionary genetics analysis version 7.0 for bigger datasets. Mol Biol Evol. 2016;33:1870–4. 10.1093/molbev/msw054 PMC8210823 27004904

[42] Ronquist F, Huelsenbeck JP. MrBayes 3: Bayesian phylogenetic inference under mixed models. Bioinformatics. 2003;19:1572–4. 10.1093/bioinformatics/btg180 12912839

[43] Lanfear R, Calcott B, Kainer D, Mayer C, Stamatakis A. Selecting optimal partitioning schemes for phylogenomic datasets. BMC Evol Biol. 2014;14:82. 10.1186/1471-2148-14-82 PMC4012149 24742000

[44] Lanfear R, Frandsen PB, Wright AM, Senfeld T, Calcott B. PartitionFinder 2: new methods for selecting partitioned models of evolution for molecular and morphological phylogenetic analyses. Mol Biol Evol. 2017;34(3):772–3. 10.1093/molbev/msw260 28013191

