# Supplementary material for: Chromosomal variation among populations of a fungus-farming ant: implications for karyotype evolution and potential restriction to gene flow
Source: BMC Evol Biol. 2018 Sep 21;18:146. doi: 10.1186/s12862-018-1247-5 (PMC6150965; doi:10.1186/s12862-018-1247-5)
Supplement: Supplementary file 1 — Table S1-S5. Results from the karyomorphometrical analyses of the Trachymyrmex holmgreni populations analyzed in the study. (DOCX 41 kb) [file 12862_2018_1247_MOESM1_ESM.docx]

**Table S1** - Karyomorphometric analyses of the chromosomes of *T. holmgreni* from Cidreira (RS) population.

| **Chromosome** | **TL(µM)** | **L(µM)** | **S(µM)** | **RL(µM)** | ***r*** | **Classification** |
| --- | --- | --- | --- | --- | --- | --- |
| 1 | 6,29±0,82 | 3,32±0,44 | 2,97±0,39 | 7,58±0,23 | 1,12±0,04 | **Metacêntrico** |
| 2 | 6,06±0,83 | 3,18±0,39 | 2,87±0,46 | 7,29±0,2 | 1,11±0,08 | **Metacêntrico** |
| 3 | 5,4±0,81 | 3,06±0,42 | 2,35±0,41 | 6,49±0,36 | 1,31±0,1 | **Metacêntrico** |
| 4 | 5,03±0,76 | 2,91±0,48 | 2,11±0,29 | 6,04±0,26 | 1,38±0,07 | **Metacêntrico** |
| 5 | 4,48±0,58 | 2,42±0,31 | 2,06±0,3 | 5,39±0,15 | 1,18±0,11 | **Metacêntrico** |
| 6 | 4,28±0,51 | 2,27±0,23 | 2,01±0,29 | 5,15±0,17 | 1,13±0,05 | **Metacêntrico** |
| 7 | 4,13±0,51 | 2,18±0,21 | 1,96±0,31 | 4,97±0,13 | 1,12±0,1 | **Metacêntrico** |
| 8 | 4,04±0,5 | 2,1±0,24 | 1,95±0,27 | 4,86±0,1 | 1,08±0,08 | **Metacêntrico** |
| 9 | 3,98±0,51 | 2,1±0,26 | 1,88±0,26 | 4,79±0,09 | 1,12±0,07 | **Metacêntrico** |
| 10 | 3,91±0,51 | 2,1±0,31 | 1,81±0,21 | 4,71±0,06 | 1,15±0,06 | **Metacêntrico** |
| 11 | 3,84±0,46 | 1,99±0,22 | 1,84±0,25 | 4,63±0,07 | 1,08±0,05 | **Metacêntrico** |
| 12 | 3,76±0,45 | 2,05±0,3 | 1,72±0,18 | 4,53±0,06 | 1,19±0,13 | **Metacêntrico** |
| 13 | 3,71±0,45 | 1,99±0,22 | 1,72±0,25 | 4,47±0,07 | 1,17±0,1 | **Metacêntrico** |
| 14 | 3,64±0,45 | 1,96±0,28 | 1,67±0,2 | 4,38±0,07 | 1,17±0,13 | **Metacêntrico** |
| 15 | 3,59±0,45 | 1,94±0,23 | 1,66±0,26 | 4,32±0,08 | 1,18±0,13 | **Metacêntrico** |
| 16 | 3,52±0,4 | 1,86±0,25 | 1,66±0,18 | 4,25±0,09 | 1,14±0,07 | **Metacêntrico** |
| 17 | 3,46±0,4 | 1,86±0,18 | 1,6±0,25 | 4,17±0,07 | 1,18±0,13 | **Metacêntrico** |
| 18 | 3,41±0,39 | 1,83±0,22 | 1,58±0,2 | 4,12±0,09 | 1,17±0,1 | **Metacêntrico** |
| 19 | 3,35±0,4 | 1,83±0,21 | 1,52±0,2 | 4,04±0,1 | 1,21±0,08 | **Metacêntrico** |
| 20 | 3,18±0,45 | 1,74±0,23 | 1,44±0,23 | 3,82±0,14 | 1,21±0,09 | **Metacêntrico** |
| **∑(KL)** | **83,06** |  |  |  |  |  |

**TL**: total length; **L**: long arm length; **S**: short arm length; **RL**: relative length; **r**: arm ratio (= L/S).

**Table S2** – Karyomorphometric analyses of the chromosomes of *T. holmgreni* from Torres (RS) population.

| **Chromosome** | **TL(µM)** | **L(µM)** | **S(µM)** | **RL(µM)** | ***r*** | **Classification** |
| --- | --- | --- | --- | --- | --- | --- |
| 1 | 6,06±0,87 | 3,19±0,44 | 2,87±0,46 | 7,33±0,35 | 1,12±0,08 | Metacentric |
| 1 | 5,77±0,81 | 3,05±0,41 | 2,71±0,41 | 6,98±0,20 | 1,13±0,08 | Metacentric |
| 2 | 5,1±0,62 | 2,94±0,4 | 2,17±0,27 | 6,19±0,22 | 1,36±0,12 | Metacentric |
| 2 | 4,92±0,59 | 2,75±0,43 | 2,17±0,22 | 5,98±0,31 | 1,27±0,14 | Metacentric |
| 3 | 4,35±0,69 | 2,35±0,42 | 2,00±0,33 | 5,26±0,19 | 1,18±0,17 | Metacentric |
| 3 | 4,23±0,66 | 2,26±0,34 | 1,97±0,33 | 5,11±0,10 | 1,15±0,09 | Metacentric |
| 4 | 4,11±0,66 | 2,22±0,36 | 1,89±0,34 | 4,96±0,13 | 1,18±0,14 | Metacentric |
| 4 | 4,05±0,63 | 2,14±0,36 | 1,91±0,30 | 4,89±0,13 | 1,12±0,08 | Metacentric |
| 5 | 3,98±0,62 | 2,12±0,32 | 1,86±0,32 | 4,81±0,08 | 1,15±0,08 | Metacentric |
| 5 | 3,92±0,61 | 2,07±0,33 | 1,85±0,29 | 4,73±0,08 | 1,12±0,07 | Metacentric |
| 6 | 3,85±0,62 | 2,11±0,37 | 1,74±0,28 | 4,64±0,10 | 1,21±0,11 | Metacentric |
| 6 | 3,77±0,61 | 2,01±0,34 | 1,76±0,30 | 4,55±0,10 | 1,15±0,13 | Metacentric |
| 7 | 3,73±0,60 | 2,01±0,30 | 1,73±0,31 | 4,51±0,08 | 1,17±0,07 | Metacentric |
| 7 | 3,70±0,58 | 1,98±0,28 | 1,72±0,32 | 4,47±0,08 | 1,16±0,10 | Metacentric |
| 8 | 3,67±0,57 | 1,93±0,34 | 1,74±0,24 | 4,43±0,08 | 1,10±0,08 | Metacentric |
| 8 | 3,60±0,56 | 1,92±0,27 | 1,68±0,29 | 4,35±0,08 | 1,15±0,06 | Metacentric |
| 9 | 3,56±0,55 | 1,85±0,32 | 1,71±0,27 | 4,3±0,07 | 1,09±0,14 | Metacentric |
| 9 | 3,50±0,53 | 1,89±0,30 | 1,61±0,25 | 4,23±0,09 | 1,18±0,12 | Metacentric |
| 10 | 3,45±0,55 | 1,89±0,38 | 1,56±0,23 | 4,17±0,11 | 1,21±0,20 | Metacentric |
| 10 | 3,40±0,54 | 1,82±0,27 | 1,58±0,30 | 4,1±0,09 | 1,16±0,11 | Metacentric |
| **∑(KL)** | **82,72** |  |  |  |  |  |

**TL**: total length; **L**: long arm length; **S**: short arm length; **RL**: relative length; **r**: arm ratio (= L/S).

**Table S3** - Karyomorphometric analyses of the chromosomes of *T. holmgreni* from Balneário Gaivota (SC) population.

| **Chromosome** | **TL(µM)** | **L(µM)** | **S(µM)** | **RL(µM)** | ***r*** | **Classification** |
| --- | --- | --- | --- | --- | --- | --- |
| 1 | 5,30±0,78 | 2,81±0,49 | 2,49±0,33 | 7,23±0,52 | 1,13±0,11 | Metacentric |
| 1 | 4,93±0,77 | 2,62±0,49 | 2,31±0,29 | 6,70±0,37 | 1,13±0,09 | Metacentric |
| 2 | 4,48±0,71 | 2,53±0,42 | 1,95±0,34 | 6,09±0,27 | 1,31±0,15 | Metacentric |
| 2 | 4,34±0,70 | 2,44±0,47 | 1,90±0,25 | 5,90±0,25 | 1,28±0,12 | Metacentric |
| 3 | 3,90±0,61 | 2,06±0,33 | 1,84±0,29 | 5,30±0,20 | 1,12±0,06 | Metacentric |
| 3 | 3,80±0,54 | 2,01±0,37 | 1,78±0,20 | 5,17±0,14 | 1,12±0,13 | Metacentric |
| 4 | 3,67±0,46 | 1,97±0,29 | 1,71±0,25 | 5,01±0,12 | 1,17±0,20 | Metacentric |
| 4 | 3,59±0,46 | 1,94±0,34 | 1,65±0,17 | 4,90±0,12 | 1,18±0,18 | Metacentric |
| 5 | 3,53±0,44 | 1,85±0,31 | 1,68±0,16 | 4,82±0,13 | 1,10±0,14 | Metacentric |
| 5 | 3,46±0,44 | 1,85±0,32 | 1,61±0,15 | 4,73±0,09 | 1,15±0,15 | Metacentric |
| 6 | 3,41±0,44 | 1,82±0,28 | 1,60±0,20 | 4,66±0,09 | 1,14±0,14 | Metacentric |
| 6 | 3,38±0,43 | 1,80±0,27 | 1,58±0,20 | 4,61±0,11 | 1,14±0,15 | Metacentric |
| 7 | 3,34±0,45 | 1,78±0,27 | 1,56±0,22 | 4,55±0,08 | 1,14±0,15 | Metacentric |
| 7 | 3,31±0,44 | 1,76±0,35 | 1,55±0,15 | 4,52±0,09 | 1,14±0,20 | Metacentric |
| 8 | 3,28±0,43 | 1,73±0,28 | 1,55±0,19 | 4,47±0,08 | 1,12±0,14 | Metacentric |
| 8 | 3,26±0,43 | 1,71±0,28 | 1,55±0,19 | 4,44±0,07 | 1,10±0,13 | Metacentric |
| 9 | 3,18±0,44 | 1,68±0,28 | 1,51±0,20 | 4,34±0,15 | 1,12±0,16 | Metacentric |
| 9 | 3,13±0,41 | 1,67±0,26 | 1,47±0,19 | 4,27±0,11 | 1,14±0,15 | Metacentric |
| 10 | 3,09±0,44 | 1,65±0,30 | 1,44±0,17 | 4,21±0,13 | 1,15±0,17 | Metacentric |
| 10 | 3,00±0,46 | 1,61±0,35 | 1,4±0,18 | 4,09±0,15 | 1,15±0,23 | Metacentric |
| **∑(KL)** | **73,38** |  |  |  |  |  |

**Table S4** – Karyomorphometric analyses of the chromosomes of *T. holmgreni* from Morro dos Conventos (SC) population.

| **Chromosome** | **TL(µM)** | **L(µM)** | **S(µM)** | **RL(µM)** | ***r*** | **Classification** |
| --- | --- | --- | --- | --- | --- | --- |
| 1 | 5,25±0,69 | 2,79±0,33 | 2,38±0,38 | 7,65±0,37 | 1,12±0,07 | Metacentric |
| 1 | 4,90±0,61 | 2,73±0,35 | 2,25±0,29 | 7,15±0,28 | 1,15±0,09 | Metacentric |
| 2 | 4,31±0,58 | 2,57±0,37 | 1,86±0,24 | 6,28±0,27 | 1,35±0,12 | Metacentric |
| 2 | 4,10±0,57 | 2,31±0,32 | 1,77±0,28 | 5,97±0,24 | 1,31±0,11 | Metacentric |
| 3 | 3,61±0,43 | 1,90±0,24 | 1,78±0,22 | 5,27±0,12 | 1,08±0,10 | Metacentric |
| 3 | 3,53±0,45 | 1,86±0,27 | 1,65±0,19 | 5,14±0,13 | 1,12±0,07 | Metacentric |
| 4 | 3,42±0,40 | 1,75±0,25 | 1,70±0,17 | 4,98±0,14 | 1,10±0,09 | Metacentric |
| 4 | 3,30±0,42 | 1,76±0,25 | 1,52±0,19 | 4,80±0,07 | 1,14±0,11 | Metacentric |
| 5 | 3,26±0,41 | 1,72±0,22 | 1,53±0,21 | 4,74±0,09 | 1,14±0,09 | Metacentric |
| 5 | 3,21±0,40 | 1,69±0,23 | 1,53±0,19 | 4,67±0,09 | 1,15±0,09 | Metacentric |
| 6 | 3,16±0,40 | 1,61±0,22 | 1,57±0,20 | 4,60±0,09 | 1,09±0,08 | Metacentric |
| 6 | 3,10±0,36 | 1,66±0,20 | 1,43±0,18 | 4,52±0,09 | 1,14±0,11 | Metacentric |
| 7 | 3,07±0,36 | 1,61±0,20 | 1,46±0,19 | 4,48±0,07 | 1,11±0,09 | Metacentric |
| 7 | 3,04±0,38 | 1,65±0,20 | 1,40±0,18 | 4,43±0,07 | 1,13±0,05 | Metacentric |
| 8 | 3,00±0,37 | 1,58±0,20 | 1,46±0,19 | 4,37±0,09 | 1,17±0,10 | Metacentric |
| 8 | 2,98±0,36 | 1,61±0,20 | 1,41±0,18 | 4,34±0,11 | 1,13±0,09 | Metacentric |
| 9 | 2,94±0,38 | 1,62±0,20 | 1,39±0,19 | 4,29±0,10 | 1,15±0,06 | Metacentric |
| 9 | 2,91±0,37 | 1,53±0,22 | 1,43±0,16 | 4,24±0,11 | 1,09±0,08 | Metacentric |
| 10 | 2,85±0,34 | 1,53±0,18 | 1,37±0,18 | 4,15±0,08 | 1,13±0,08 | Metacentric |
| 10 | 2,70±0,39 | 1,44±0,21 | 1,29±0,19 | 3,92±0,14 | 1,09±0,08 | Metacentric |
| **∑(KL)** | **68,63** |  |  |  |  |  |

**TL**: total length; **L**: long arm length; **S**: short arm length; **RL**: relative length; **r**: arm ratio (= L/S).

**Table S5** – Karyomorphometric analyses of the chromosomes of *T. holmgreni* from Cachoeira do Campo (MG) population.

| **Chromosome** | **TL(µM)** | **L(µM)** | **S(µM)** | **RL(µM)** | ***r*** | **Classification** |
| --- | --- | --- | --- | --- | --- | --- |
| 1 | 4,87±0,60 | 2,59±0,31 | 2,22±0,30 | 7,50±0,29 | 1,16±0,05 | Metacentric |
| 1 | 4,58±0,63 | 2,44±0,32 | 2,18±0,33 | 7,09±0,33 | 1,10±0,07 | Metacentric |
| 2 | 4,17±0,41 | 2,31±0,26 | 1,76±0,19 | 6,14±0,17 | 1,30±0,14 | Metacentric |
| 2 | 3,80±0,37 | 2,19±0,21 | 1,61±0,20 | 5,80±0,14 | 1,30±0,12 | Metacentric |
| 3 | 3,42±0,48 | 1,78±0,33 | 1,64±0,18 | 5,23±0,18 | 1,09±0,13 | Metacentric |
| 3 | 3,34±0,40 | 1,77±0,24 | 1,57±0,17 | 5,13±0,11 | 1,14±0,07 | Metacentric |
| 4 | 3,28±0,31 | 1,74±0,16 | 1,55±0,16 | 4,97±0,08 | 1,12±0,06 | Metacentric |
| 4 | 3,21±0,32 | 1,68±0,21 | 1,51±0,13 | 4,88±0,09 | 1,11±0,09 | Metacentric |
| 5 | 3,20±0,32 | 1,68±0,14 | 1,50±0,19 | 4,76±0,09 | 1,12±0,09 | Metacentric |
| 5 | 3,14±0,29 | 1,66±0,18 | 1,44±0,13 | 4,67±0,10 | 1,13±0,09 | Metacentric |
| 6 | 3,07±0,28 | 1,64±0,16 | 1,44±0,14 | 4,61±0,09 | 1,09±0,07 | Metacentric |
| 6 | 3,04±0,29 | 1,63±0,13 | 1,41±0,17 | 4,58±0,09 | 1,16±0,08 | Metacentric |
| 7 | 3,00±0,28 | 1,60±0,18 | 1,36±0,11 | 4,50±0,07 | 1,15±0,08 | Metacentric |
| 7 | 2,99±0,29 | 1,56±0,16 | 1,37±0,15 | 4,48±0,07 | 1,09±0,07 | Metacentric |
| 8 | 2,96±0,28 | 1,54±0,16 | 1,43±0,14 | 4,45±0,06 | 1,10±0,07 | Metacentric |
| 8 | 2,92±0,29 | 1,53±0,18 | 1,38±0,11 | 4,40±0,06 | 1,12±0,05 | Metacentric |
| 9 | 2,89±0,29 | 1,57±0,17 | 1,30±0,14 | 4,35±0,06 | 1,20±0,09 | Metacentric |
| 9 | 2,80±0,26 | 1,49±0,16 | 1,29±0,12 | 4,24±0,07 | 1,16±0,07 | Metacentric |
| 10 | 2,77±0,27 | 1,56±0,14 | 1,22±0,16 | 4,21±0,08 | 1,24±0,11 | Metacentric |
| 10 | 2,62±0,25 | 1,41±0,15 | 1,23±0,11 | 4,01±0,14 | 1,19±0,08 | Metacentric |
| **∑(KL)** | **66,08** |  |  |  |  |  |

**TL**: total length; **L**: long arm length; **S**: short arm length; **RL**: relative length; **r**: arm ratio (= L/S).
