# Supplementary figures and images for: Chromosomal variation among populations of a fungus-farming ant: implications for karyotype evolution and potential restriction to gene flow
Source: BMC Evol Biol. 2018 Sep 21;18:146. doi: 10.1186/s12862-018-1247-5 (PMC6150965; doi:10.1186/s12862-018-1247-5)

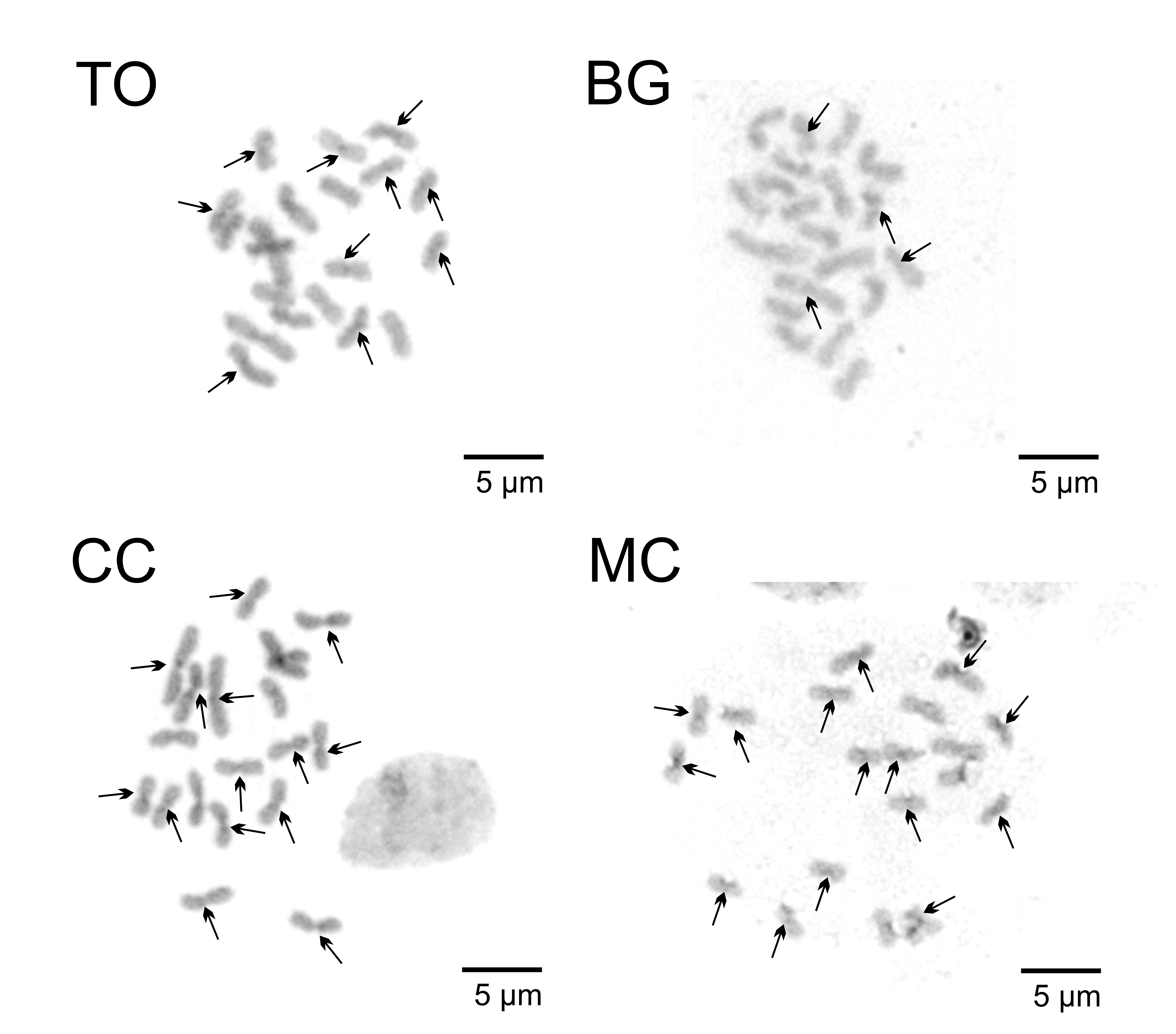

Supplement: Supplementary file 2 — Figure S1. C-banding in worker metaphases of Trachymyrmex holmgreni populations. (a) TO – Torres (RS), (b) MC – Morro dos Conventos (SC), and (c) Cachoeira do Campo (MG). The arrows point to dark grey heterochromatin blocks. Scale bar = 5 μm. C-banding was not done in the Cidreira population. (TIF 4265 kb) [file 12862_2018_1247_MOESM2_ESM.tif]

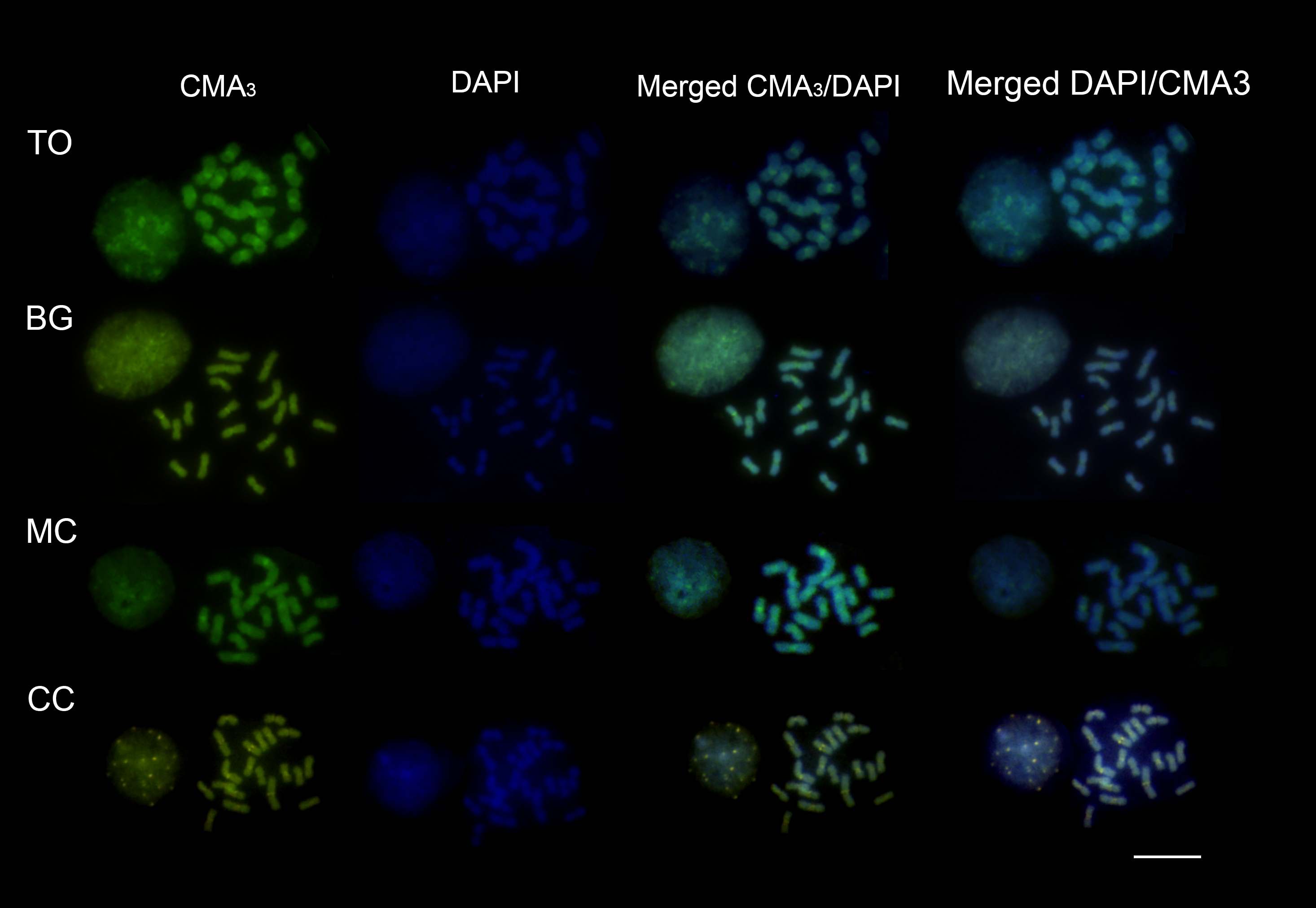

Supplement: Supplementary file 3 — Figure S2. Fluorochrome-stained metaphases of Trachymyrmex holmgreni from the four studied populations: by columns: CMA3, DAPI, merged CMA3 over DAPI and merged DAPI over CMA3. By rows: (a) TO – Torres, (b) BG – Balneário Gaivota, (c) MC – Morro dos Conventos, and (d) CC – Cachoeira do Campo. Positive GC-rich blocks were observed in all chromosome pairs at the centromere, as represented in the ideograms. Scale bar = 5 μm. Fluorochrome-staining was not done in the Cidreira population. (TIF 6249 kb) [file 12862_2018_1247_MOESM3_ESM.tif]
