# Supplementary material for: Chromosomal variation among populations of a fungus-farming ant: implications for karyotype evolution and potential restriction to gene flow
Source: BMC Evol Biol. 2018 Sep 21;18:146. doi: 10.1186/s12862-018-1247-5 (PMC6150965; doi:10.1186/s12862-018-1247-5)
Supplement: Supplementary file 5 — Table S6. Karyomorphometrical analyses of the specimens of Trachymyrmex holmgreni from the five populations analyzed in the study. ∑TL: total length; KL mean karyotype length (= ∑TL/2n) ± SD: standard deviation; CV coefficient of variation (= ± SD/KL). All measurements are given in “μM”. (DOCX 57 kb) [file 12862_2018_1247_MOESM5_ESM.docx]

**Table S6** **–** Karyomorphometrical analyses of the specimens of *Trachymyrmex holmgreni* from the five populations analyzed in this study. ∑TL: total length; KL mean karyotype length (= ∑TL/2n) ± SD: standard deviation; CV coefficient of variation (= ±SD/KL). All measurements are given in “μM”.

| Population | CI – Rio Grande do Sul |  |  |  |  |  |  |  |  |  |  |
| --- | --- | --- | --- | --- | --- | --- | --- | --- | --- | --- | --- |
|  |  | Specimens | | | | | | | | | |
|  |  | 1 | 2 | 3 | 4 | 5 | 6 | 7 | 8 | 9 | 10 |
| Chromosomes | 1 | 5,89 | 5,98 | 5,94 | 5,93 | 5,35 | 6,92 | 5,56 | 7,09 | 7,09 | 8,03 |
|  | 1 | 5,74 | 5,89 | 5,92 | 5,65 | 4,89 | 6,78 | 5,51 | 6,33 | 6,33 | 7,96 |
|  | 2 | 5,21 | 5,69 | 4,79 | 4,80 | 4,42 | 6,08 | 5,00 | 6,00 | 6,00 | 7,10 |
|  | 2 | 4,53 | 4,76 | 4,59 | 4,61 | 4,42 | 5,92 | 4,85 | 5,17 | 5,17 | 6,78 |
|  | 3 | 4,49 | 4,18 | 4,01 | 4,27 | 3,74 | 4,91 | 4,13 | 4,86 | 4,86 | 5,76 |
|  | 3 | 4,12 | 3,97 | 3,83 | 4,16 | 3,58 | 4,76 | 3,96 | 4,72 | 4,72 | 5,29 |
|  | 4 | 4,01 | 3,93 | 3,79 | 4,09 | 3,55 | 4,73 | 3,77 | 4,13 | 4,13 | 5,28 |
|  | 4 | 3,99 | 3,89 | 3,75 | 3,86 | 3,46 | 4,58 | 3,65 | 4,04 | 4,04 | 5,18 |
|  | 5 | 3,87 | 3,88 | 3,75 | 3,75 | 3,38 | 4,48 | 3,56 | 4,01 | 4,01 | 5,18 |
|  | 5 | 3,72 | 3,81 | 3,63 | 3,70 | 3,32 | 4,37 | 3,55 | 3,99 | 3,99 | 5,14 |
|  | 6 | 3,63 | 3,77 | 3,58 | 3,68 | 3,29 | 4,19 | 3,54 | 3,96 | 3,96 | 4,95 |
|  | 6 | 3,54 | 3,58 | 3,52 | 3,61 | 3,26 | 4,14 | 3,51 | 3,92 | 3,92 | 4,84 |
|  | 7 | 3,53 | 3,56 | 3,36 | 3,54 | 3,18 | 4,07 | 3,50 | 3,86 | 3,86 | 4,77 |
|  | 7 | 3,51 | 3,48 | 3,30 | 3,49 | 3,18 | 4,00 | 3,26 | 3,83 | 3,83 | 4,68 |
|  | 8 | 3,42 | 3,38 | 3,26 | 3,49 | 3,12 | 3,93 | 3,26 | 3,77 | 3,77 | 4,67 |
|  | 8 | 3,34 | 3,33 | 3,26 | 3,33 | 3,12 | 3,92 | 3,21 | 3,72 | 3,72 | 4,41 |
|  | 9 | 3,27 | 3,27 | 3,24 | 3,23 | 3,06 | 3,90 | 3,19 | 3,58 | 3,58 | 4,37 |
|  | 9 | 3,24 | 3,22 | 3,22 | 3,20 | 3,05 | 3,82 | 3,14 | 3,47 | 3,47 | 4,32 |
|  | 10 | 3,17 | 3,19 | 3,19 | 3,19 | 2,91 | 3,82 | 3,02 | 3,33 | 3,33 | 4,26 |
|  | 10 | 2,79 | 3,07 | 3,00 | 3,01 | 2,80 | 3,75 | 2,82 | 3,26 | 3,26 | 4,18 |
|  | **Σ_TL_** | 79,03 | 79,83 | 76,94 | 78,58 | 71,10 | 93,09 | 75,98 | 87,03 | 81,84 | 107,15 |
|  | **KL** | 3,95 | 3,99 | 3,85 | 3,93 | 3,56 | 4,65 | 3,80 | 4,35 | 4,35 | 5,36 |
|  | **SD** | 0,84 | 0,89 | 0,84 | 0,79 | 0,69 | 0,99 | 0,81 | 1,05 | 1,05 | 1,18 |
|  | **CV** | 0,21 | 0,22 | 0,22 | 0,20 | 0,19 | 0,21 | 0,21 | 0,24 | 0,24 | 0,22 |
| Population | TO – Rio Grande do Sul |  |  |  |  |  |  |  |  |  |  |
|  |  | 1 | 2 | 3 | 4 | 5 | 6 | 7 | 8 | 9 | 10 |
| Chromosomes | 1 | 6,34 | 6,83 | 6,59 | 7,42 | 6,52 | 5,88 | 4,62 | 4,79 | 5,80 | 5,78 |
|  | 1 | 5,77 | 6,72 | 5,89 | 7,01 | 6,43 | 5,82 | 4,59 | 4,71 | 5,53 | 5,18 |
|  | 2 | 4,89 | 5,59 | 5,48 | 6,25 | 5,52 | 5,09 | 4,22 | 4,29 | 4,95 | 4,76 |
|  | 2 | 4,86 | 5,47 | 5,37 | 6,12 | 4,99 | 4,70 | 4,20 | 4,26 | 4,63 | 4,60 |
|  | 3 | 4,42 | 5,13 | 4,61 | 5,34 | 4,95 | 4,20 | 3,40 | 3,50 | 4,38 | 3,60 |
|  | 3 | 4,31 | 4,85 | 4,58 | 5,29 | 4,75 | 4,20 | 3,28 | 3,45 | 4,05 | 3,56 |
|  | 4 | 4,24 | 4,80 | 4,44 | 5,16 | 4,66 | 3,93 | 3,26 | 3,34 | 3,82 | 3,45 |
|  | 4 | 4,23 | 4,62 | 4,42 | 5,11 | 4,51 | 3,91 | 3,23 | 3,22 | 3,82 | 3,44 |
|  | 5 | 3,94 | 4,58 | 4,38 | 5,02 | 4,49 | 3,85 | 3,22 | 3,21 | 3,77 | 3,34 |
|  | 5 | 3,79 | 4,37 | 4,33 | 4,98 | 4,46 | 3,83 | 3,16 | 3,14 | 3,77 | 3,34 |
|  | 6 | 3,62 | 4,35 | 4,26 | 4,91 | 4,46 | 3,80 | 3,06 | 3,13 | 3,60 | 3,28 |
|  | 6 | 3,58 | 4,27 | 4,26 | 4,76 | 4,28 | 3,78 | 2,95 | 3,07 | 3,58 | 3,16 |
|  | 7 | 3,58 | 4,26 | 4,11 | 4,73 | 4,27 | 3,74 | 2,92 | 3,05 | 3,55 | 3,12 |
|  | 7 | 3,55 | 4,24 | 4,08 | 4,63 | 4,19 | 3,74 | 2,91 | 3,03 | 3,53 | 3,11 |
|  | 8 | 3,51 | 4,15 | 4,04 | 4,61 | 4,18 | 3,73 | 2,91 | 3,03 | 3,44 | 3,09 |
|  | 8 | 3,42 | 4,05 | 4,00 | 4,53 | 4,09 | 3,63 | 2,87 | 2,95 | 3,43 | 3,06 |
|  | 9 | 3,39 | 3,95 | 3,91 | 4,52 | 4,09 | 3,55 | 2,87 | 2,91 | 3,37 | 3,05 |
|  | 9 | 3,26 | 3,91 | 3,84 | 4,41 | 4,01 | 3,49 | 2,84 | 2,90 | 3,28 | 3,01 |
|  | 10 | 3,24 | 3,91 | 3,84 | 4,40 | 3,95 | 3,49 | 2,79 | 2,81 | 3,12 | 3,00 |
|  | 10 | 3,23 | 3,90 | 3,78 | 4,35 | 3,81 | 3,39 | 2,77 | 2,76 | 3,11 | 2,88 |
|  | **Σ_TL_** | **81,16** | **93,95** | **90,21** | **103,56** | **92,62** | **81,75** | **66,07** | **67,55** | **78,54** | **71,83** |
|  | **KL** | **4,06** | **4,70** | **4,51** | **5,18** | **4,63** | **4,09** | **3,30** | **3,38** | **3,93** | **3,59** |
|  | **SD** | **0,86** | **0,87** | **0,75** | **0,87** | **0,75** | **0,73** | **0,60** | **0,62** | **0,76** | **0,82** |
|  | **CV** | **0,21** | **0,18** | **0,17** | **0,17** | **0,16** | **0,18** | **0,18** | **0,18** | **0,19** | **0,23** |
| Population | BG – Santa Catarina |  |  |  |  |  |  |  |  |  |  |
|  |  | 1 | 2 | 3 | 4 | 5 | 6 | 7 | 8 | 9 | 10 |
| Chromosomes | 1 | 5,75 | 5,62 | 3,89 | 4,69 | 4,87 | 4,72 | 5,63 | 5,15 | 5,75 | 6,39 |
|  | 1 | 6,27 | 5,25 | 3,44 | 4,23 | 4,13 | 4,59 | 5,28 | 5,16 | 4,70 | 5,81 |
|  | 2 | 5,51 | 4,60 | 3,21 | 4,38 | 3,50 | 4,23 | 5,12 | 4,24 | 5,51 | 4,95 |
|  | 2 | 5,61 | 4,65 | 3,33 | 3,93 | 3,48 | 4,02 | 4,84 | 4,19 | 4,90 | 4,90 |
|  | 3 | 4,28 | 4,24 | 2,81 | 3,26 | 2,79 | 3,57 | 4,61 | 3,88 | 4,20 | 3,82 |
|  | 3 | 4,25 | 3,71 | 2,64 | 3,31 | 2,92 | 3,68 | 4,48 | 3,94 | 4,61 | 3,97 |
|  | 4 | 4,48 | 4,14 | 3,03 | 3,11 | 3,06 | 3,64 | 4,04 | 3,83 | 3,84 | 4,00 |
|  | 4 | 4,36 | 3,91 | 2,87 | 3,11 | 3,15 | 3,65 | 3,81 | 3,56 | 3,85 | 3,61 |
|  | 5 | 3,79 | 3,58 | 2,66 | 2,92 | 3,07 | 3,19 | 4,03 | 3,40 | 3,57 | 3,55 |
|  | 5 | 3,79 | 3,80 | 2,41 | 2,77 | 2,79 | 3,29 | 3,82 | 3,72 | 3,68 | 3,73 |
|  | 6 | 4,15 | 3,56 | 2,88 | 3,25 | 2,65 | 3,13 | 3,90 | 3,44 | 3,95 | 3,60 |
|  | 6 | 3,92 | 3,64 | 2,90 | 3,14 | 2,77 | 3,41 | 3,85 | 3,26 | 3,60 | 3,45 |
|  | 7 | 3,91 | 3,71 | 2,64 | 2,64 | 2,64 | 3,17 | 3,59 | 3,30 | 3,70 | 3,45 |
|  | 7 | 3,82 | 3,57 | 2,53 | 2,98 | 2,68 | 3,33 | 3,67 | 3,33 | 3,83 | 3,43 |
|  | 8 | 3,68 | 3,55 | 2,53 | 2,99 | 2,53 | 3,47 | 3,35 | 3,21 | 3,79 | 3,43 |
|  | 8 | 4,05 | 3,67 | 2,76 | 2,98 | 2,45 | 3,23 | 4,11 | 3,33 | 3,65 | 3,34 |
|  | 9 | 3,93 | 3,35 | 2,89 | 3,00 | 2,93 | 3,22 | 3,90 | 3,11 | 3,82 | 3,38 |
|  | 9 | 3,71 | 3,55 | 2,77 | 2,88 | 2,67 | 3,22 | 3,35 | 3,13 | 3,54 | 3,22 |
|  | 10 | 3,61 | 3,19 | 2,41 | 2,90 | 2,36 | 2,90 | 3,36 | 3,19 | 3,70 | 3,25 |
|  | 10 | 3,57 | 3,35 | 2,57 | 2,76 | 2,22 | 3,13 | 3,37 | 3,01 | 3,76 | 3,24 |
|  | **Σ_TL_** | **86,43** | **78,65** | **57,15** | **65,23** | **59,66** | **70,78** | **82,10** | **73,38** | **81,97** | **78,55** |
|  | **KL** | **4,32** | **3,93** | **2,86** | **3,26** | **2,98** | **3,54** | **4,11** | **3,67** | **4,10** | **3,93** |
|  | **SD** | **0,80** | **0,64** | **0,37** | **0,58** | **0,62** | **0,50** | **0,68** | **0,62** | **0,65** | **0,89** |
|  | **CV** | **0,19** | **0,16** | **0,13** | **0,18** | **0,21** | **0,14** | **0,16** | **0,17** | **0,16** | **0,23** |
| Population | MC – Santa Catarina |  |  |  |  |  |  |  |  |  |  |
|  |  | 1 | 2 | 3 | 4 | 5 | 6 | 7 | 8 | 9 | 10 |
| Chromosomes | 1 | 4,92 | 5,43 | 4,80 | 4,51 | 4,71 | 4,27 | 6,01 | 5,73 | 5,60 | 6,39 |
|  | 1 | 5,03 | 5,13 | 4,55 | 4,41 | 4,18 | 3,96 | 5,42 | 5,51 | 5,14 | 5,81 |
|  | 2 | 4,28 | 4,52 | 3,74 | 4,02 | 3,77 | 3,31 | 4,74 | 5,14 | 4,60 | 4,95 |
|  | 2 | 3,75 | 4,41 | 3,79 | 4,03 | 3,54 | 3,10 | 4,48 | 4,80 | 4,29 | 4,90 |
|  | 3 | 3,46 | 3,64 | 3,53 | 3,47 | 3,15 | 2,80 | 4,26 | 3,85 | 3,90 | 3,82 |
|  | 3 | 3,43 | 3,46 | 3,39 | 3,42 | 2,99 | 2,74 | 4,19 | 3,93 | 3,82 | 3,97 |
|  | 4 | 3,39 | 3,39 | 3,34 | 3,25 | 2,94 | 2,55 | 3,96 | 3,59 | 3,51 | 4,00 |
|  | 4 | 3,09 | 3,36 | 3,03 | 3,23 | 2,80 | 2,44 | 3,88 | 3,75 | 3,53 | 3,61 |
|  | 5 | 3,14 | 3,37 | 3,03 | 3,18 | 2,60 | 2,63 | 3,85 | 3,62 | 3,56 | 3,55 |
|  | 5 | 2,87 | 3,20 | 2,89 | 3,34 | 2,74 | 2,36 | 3,48 | 3,55 | 3,42 | 3,73 |
|  | 6 | 3,07 | 3,26 | 3,01 | 3,00 | 2,65 | 2,59 | 3,70 | 3,49 | 3,61 | 3,60 |
|  | 6 | 2,95 | 3,21 | 2,81 | 3,07 | 2,59 | 2,49 | 3,39 | 3,27 | 3,42 | 3,45 |
|  | 7 | 2,93 | 3,10 | 2,81 | 3,14 | 2,65 | 2,32 | 3,41 | 3,13 | 3,47 | 3,45 |
|  | 7 | 2,89 | 3,22 | 2,91 | 2,93 | 2,60 | 2,32 | 3,44 | 3,13 | 3,27 | 3,43 |
|  | 8 | 2,72 | 3,04 | 2,85 | 3,01 | 2,61 | 2,49 | 3,50 | 3,39 | 3,31 | 3,43 |
|  | 8 | 2,67 | 3,13 | 2,91 | 2,94 | 2,49 | 2,53 | 3,49 | 3,31 | 3,27 | 3,34 |
|  | 9 | 2,71 | 3,07 | 2,74 | 3,00 | 2,62 | 2,26 | 3,42 | 3,21 | 3,45 | 3,38 |
|  | 9 | 2,75 | 3,09 | 2,72 | 3,01 | 2,47 | 2,21 | 3,70 | 3,03 | 3,32 | 3,22 |
|  | 10 | 2,55 | 3,07 | 2,83 | 2,78 | 2,38 | 2,24 | 3,26 | 3,08 | 3,05 | 3,25 |
|  | 10 | 2,70 | 2,90 | 2,41 | 2,58 | 2,46 | 1,96 | 3,10 | 2,87 | 2,99 | 3,24 |
|  | **Σ_TL_** | **65,30** | **71,00** | **64,09** | **66,33** | **58,94** | **53,57** | **78,65** | **75,40** | **74,51** | **78,55** |
|  | **KL** | **3,26** | **3,55** | **3,20** | **3,32** | **2,95** | **2,68** | **3,93** | **3,77** | **3,73** | **3,93** |
|  | **SD** | **0,72** | **0,72** | **0,61** | **0,53** | **0,63** | **0,58** | **0,75** | **0,85** | **0,68** | **0,89** |
|  | **CV** | **0,22** | **0,20** | **0,19** | **0,16** | **0,21** | **0,22** | **0,19** | **0,22** | **0,18** | **0,23** |
| Population | CC –Minas Gerais |  |  |  |  |  |  |  |  |  |  |
|  |  | 1 | 2 | 3 | 4 | 5 | 6 | 7 | 8 | 9 | 10 |
| Chromosomes | 1 | 5,79 | 4,72 | 6,12 | 4,08 | 4,57 | 4,90 | 5,31 | 4,58 | 4,90 | 4,84 |
|  | 1 | 5,61 | 4,64 | 5,94 | 3,74 | 4,38 | 4,60 | 4,84 | 4,43 | 4,42 | 4,57 |
|  | 2 | 4,61 | 3,43 | 4,70 | 3,27 | 3,65 | 3,63 | 4,23 | 4,22 | 4,19 | 4,15 |
|  | 2 | 4,07 | 3,36 | 4,63 | 3,47 | 3,48 | 3,69 | 4,04 | 3,92 | 3,77 | 3,79 |
|  | 3 | 4,12 | 3,27 | 4,53 | 2,80 | 3,09 | 3,22 | 3,56 | 3,29 | 3,21 | 3,34 |
|  | 3 | 4,12 | 3,06 | 4,12 | 2,92 | 3,00 | 3,07 | 3,61 | 3,29 | 3,17 | 3,61 |
|  | 4 | 3,48 | 3,16 | 3,95 | 2,92 | 2,91 | 3,24 | 3,16 | 3,20 | 3,29 | 3,04 |
|  | 4 | 3,58 | 3,11 | 3,93 | 2,93 | 2,91 | 3,12 | 3,21 | 3,34 | 3,33 | 3,20 |
|  | 5 | 3,46 | 3,02 | 3,74 | 2,79 | 2,73 | 2,85 | 3,41 | 3,14 | 3,34 | 3,27 |
|  | 5 | 3,54 | 3,18 | 3,63 | 2,53 | 2,68 | 2,81 | 3,13 | 3,40 | 3,20 | 3,27 |
|  | 6 | 3,28 | 2,94 | 3,53 | 2,60 | 2,73 | 2,63 | 3,20 | 3,07 | 3,16 | 3,13 |
|  | 6 | 3,55 | 2,92 | 3,54 | 2,80 | 2,72 | 2,59 | 3,14 | 2,93 | 3,20 | 3,06 |
|  | 7 | 3,29 | 2,92 | 3,57 | 2,72 | 2,56 | 2,73 | 3,09 | 2,91 | 3,06 | 3,07 |
|  | 7 | 3,44 | 2,91 | 3,61 | 2,80 | 2,59 | 2,72 | 2,93 | 2,82 | 2,93 | 2,92 |
|  | 8 | 3,35 | 2,87 | 3,40 | 2,64 | 2,67 | 2,79 | 2,99 | 2,76 | 3,07 | 3,00 |
|  | 8 | 3,14 | 2,75 | 3,54 | 2,62 | 2,66 | 2,66 | 3,08 | 2,87 | 2,81 | 3,06 |
|  | 9 | 3,35 | 2,72 | 3,38 | 2,68 | 2,39 | 2,72 | 2,69 | 2,91 | 2,82 | 2,79 |
|  | 9 | 3,11 | 2,77 | 3,49 | 2,61 | 2,47 | 2,61 | 2,89 | 2,93 | 3,02 | 3,08 |
|  | 10 | 3,28 | 3,81 | 3,27 | 2,66 | 2,73 | 2,70 | 2,87 | 2,81 | 2,92 | 2,79 |
|  | 10 | 2,93 | 3,85 | 3,54 | 2,59 | 2,51 | 2,73 | 2,97 | 2,55 | 2,66 | 2,59 |
|  | **Σ**_TL_ | 75,09 | 65,40 | 80,16 | 58,16 | 59,41 | 62,02 | 68,34 | 65,36 | 66,48 | 66,58 |
|  | KL | 3,75 | 3,27 | 4,01 | 2,91 | 2,97 | 3,10 | 3,42 | 3,27 | 3,32 | 3,33 |
|  | SD | 0,78 | 0,57 | 0,81 | 0,42 | 0,60 | 0,65 | 0,69 | 0,58 | 0,57 | 0,59 |
|  | CV | 0,21 | 0,18 | 0,20 | 0,14 | 0,20 | 0,21 | 0,20 | 0,18 | 0,17 | 0,18 |
